# Association of respiratory health with occupational exposures in the Burden of Obstructive Lung Disease (BOLD) cohort: a multinational longitudinal study

**DOI:** 10.1136/bmjresp-2026-004213

**Published:** 2026-07-24

**Authors:** Valentina Quintero-Santofimio, Jixuan Ma, James Potts, Hans Kromhout, Johanna Feary, Christer Janson, Magnus Svartengren, Mahesh Padukudru Anand, Rain Jõgi, Thorarinn Gíslason, Sanjay Kamlakar Juvekar, Rune Nielsen, Gregory Erhabor, Imed Harrabi, Graham Devereux, Andrei Malinovschi, Dhiraj Agarwal, Rana Ahmed, Vanessa Garcia-Larsen, Asaad Nafees, Peter Burney, Andre F S Amaral, on behalf of the BOLD Collaborative Research Group, Hasan Hafizi, Anila Aliko, Donika Bardhi, Holta Tafa, Natasha Thanasi, Arian Mezini, Alma Teferici, Dafina Todri, Jolanda Nikolla, Rezarta Kazasi, Hamid Hacene Cherkaski, Amira Bengrait, Tabarek Haddad, Ibtissem Zgaoula, Maamar Ghit, Abdelhamid Roubhia, Soumaya Boudra, Feryal Atoui, Randa Yakoubi, Rachid Benali, Abdelghani Bencheikh, Nadia Ait-Khaled, Christine Jenkins, Guy Marks, Tessa Bird, Paola Espinel, Kate Hardaker, Brett Toelle, Michael Studnicka, Torkil Dawes, Bernd Lamprecht, Lea Schirhofer, Herve Lawin, Arsene Kpangon, Karl Kpossou, Gildas Agodokpessi, Paul Ayelo, Benjamin Fayomi, Rolus Atrokpo, Gaston Hounton, Dieudonnè Yadjodo, Bertrand Mbatchou, Atongno Humphrey Ashu, Wan C Tan, Wen Wang, NanShan Zhong, Shengming Liu, Jiachun Lu, Pixin Ran, Dali Wang, Jin-ping Zheng, Yumin Zhou, Rain Jõgi, Hendrik Laja, Katrin Ulst, Vappu Zobel, Toomas-Julius Lill, Katrin Kiili, Ira Laanelepp, Tobias Welte, Isabelle Bodemann, Henning Geldmacher, Alexandra Schweda-Linow, Thorarinn Gislason, Bryndis Benedikdtsdottir, Kristin Jörundsdottir, Lovisa Gudmundsdottir, Sigrun Gudmundsdottir, Gunnar Gudmundsson, Elin Helga Thorarinsdottir, Hjördis Sigrun Pálsdottir, Mahesh Padukudru Anand, Parvaiz A Koul, Sajjad Malik, Nissar A Hakim, Umar Hafiz Khan, Rohini Chowgule, Vasant Shetye, Jonelle Raphael, Rosel Almeda, Mahesh Tawde, Rafiq Tadvi, Sunil Katkar, Milind Kadam, Rupesh Dhanawade, Umesh Ghurup, Sanjay Juvekar, Siddhi Hirve, Somnath Sambhudas, Bharat Chaidhary, Meera Tambe, Savita Pingale, Arati Umap, Archana Umap, Nitin Shelar, Sampada Devchakke, Sharda Chaudhary, Suvarna Bondre, Savita Walke, Ashleshsa Gawhane, Anil Sapkal, Rupali Argade, Vijay Gaikwad, Dhiraj Agrawal, Babu Pawar, Shalan Mhetre, Namdev Kale, Shirish Kathale, Sundeep Salvi, Bill Brashier, Jyoti Londhe, Sapna Madas, Althea Aquart-Stewart, Akosua Francia Aikman, Talant M Sooronbaev, Bermet M Estebesova, Meerim Akmatalieva, Saadat Usenbaeva, Jypara Kydyrova, Eliza Bostonova, Ulan Sheraliev, Nuridin Marajapov, Nurgul Toktogulova, Berik Emilov, Toktogul Azilova, Gulnara Beishekeeva, Nasyikat Dononbaeva, Aijamal Tabyshova, Kevin Mortimer, Wezzie Nyapigoti, Ernest Mwangoka, Mayamiko Kambwili, Martha Chipeta, Gloria Banda, Suzgo Mkandawire, Justice Banda, Graham Devereux, Jamie Rylance, Martin Njoroge, Catherine Chirwa, Chifundo Mhango, Edgar Ngwira, Faith Zumazuma, Frank Jonas, Patrick Mjojo, Li-Cher Loh, Abdul Rashid, Siti Sholehah, Mohamed C Benjelloun, Chakib Nejjari, Mohamed Elbiaze, Karima El Rhazi, Manelle Rjimati, Btissame ElHarche, Reda Benjelloun, Yassin Chefchaou, E F M Wouters, G J Wesseling, Daniel Obaseki, Gregory Erhabor, Olayemi Awopeju, Olufemi Adewole, Amund Gulsvik, Tina Endresen, Lene Svendsen, Rune Nielsen, Marit Aardal, Hildegunn B Fleten, Gerd Eli Dale, Eli Nordeide, Malin P Grøttveit, Åsa Skjelde, Ane Aamli Gagnat, Anders Ørskov Rotevatn, Marta Erdal, Asaad A Nafees, Muhammad Irfan, Hasan Nawaz Tahir, Muhammad Noman, Roman Ul Haq, Luisito F Idolor, Teresita S de Guia, Norberto A Francisco, Camilo C Roa, Fernando G Ayuyao, Cecil Z Tady, Daniel T Tan, Sylvia Banal-Yang, Vincent M Balanag, Maria Teresita N Reyes, Renato B Dantes, Stefanni Nonna M Paraguas, Lourdes Amarillo, Lakan U Berratio, Lenora C Fernandez, Gerard S Garcia, Sullian S Naval, Thessa Reyes, Ma Flordeliza Sanchez, Leander P Simpao, Ewa Nizankowska-Mogilnicka, Jakub Frey, Rafal Harat, Filip Mejza, Pawel Nastalek, Andrzej Pajak, Wojciech Skucha, Andrzej Szczeklik, Magda Twardowska, Cristina Bárbara, Fátima Rodrigues, Hermínia Dias, João Cardoso, João Almeida, Maria João Matos, Paula Simão, Moutinho Santos, Reis Ferreira, M Al Ghobain, H Alorainy, E El-Hamad, M Al Hajjaj, A Hashi, R Dela, R Fanuncio, E Doloriel, I Marciano, L Safia, Eric Bateman, Anamika Jithoo, Desiree Adams, Edward Barnes, Jasper Freeman, Anton Hayes, Sipho Hlengwa, Christine Johannisen, Mariana Koopman, Innocentia Louw, Ina Ludick, Alta Olckers, Johanna Ryck, Janita Storbeck, Richard van Zyl-Smit, Kirthi Gunasekera, Rajitha Wickremasinghe, Asma Elsony, Hana A Elsadig, Nada Bakery Osman, Bandar Salah Noory, Monjda Awad Mohamed

**Affiliations:** 1 National Heart and Lung Institute, Imperial College London, London, UK; 2 Department of Occupational and Environmental Health, School of Public Health, Tongji Medical College, Huazhong University of Science and Technology, Wuhan, Hubei, China; 3 Institute for Risk Assessment Sciences, University of Utrecht, Utrecht, Netherlands; 4 Department of Medical Sciences, Respiratory, Allergy and Sleep Research, Uppsala University, Uppsala, Sweden; 5 Department of Medical Sciences, Occupational and Environmental Medicine, Uppsala University Hospital, Uppsala University, Uppsala, Sweden; 6 Department of Respiratory Medicine, JSS AHER, JSS Medical College, Mysore, Karnataka, India; 7 Lung Clinic, Tartu University Hospital, Tartu, Estonia; 8 Faculty of Medicine, University of Iceland, Reykjavík, Iceland; 9 Department of Sleep, Landspítali - The National University Hospital of Iceland, Reykjavík, Iceland; 10 Vadu Rural Health Program, KEM Hospital Pune Research Centre, Pune, India; 11 Dr. D.Y. Patil Medical College, Hospital and Research Centre, Dr. D.Y. Patil Vidyapeeth, Pune, India; 12 Department of Clinical Science, University of Bergen, Bergen, Norway; 13 Department of Thoracic Medicine, Haukeland University Hospital, Bergen, Norway; 14 Department of Medicine, Obafemi Awolowo University/Obafemi Awolowo University Teaching Hospitals Complex, Ife, Osun, Nigeria; 15 Ibn El Jazzar Faculty of Medicine of Sousse, University of Sousse, Sousse, Tunisia; 16 Clinical Sciences, Liverpool School of Tropical Medicine, Liverpool, UK; 17 Department of Medical Sciences, Clinical Physiology, Uppsala University, Uppsala, Sweden; 18 The Epidemiological Laboratory (Epi-Lab), Khartoum, Sudan; 19 Centre for Chronic Disease and Population Health Research, School of Population Health, RCSI University of Medicine and Health Sciences, Dublin, Ireland; 20 Department of International Health, Bloomberg School of Public Health, Johns Hopkins University, Baltimore, Maryland, USA; 21 Department of Community Health Sciences, Aga Khan University, Karachi, Pakistan

**Keywords:** COPD epidemiology, Occupational Lung Disease

## Abstract

**Background:**

Occupational exposures are contributors to chronic respiratory diseases, particularly in low- and middle-income countries (LMICs), where regulatory policies are limited. We investigated associations between occupational exposures and respiratory outcomes using longitudinal data from the Burden of Obstructive Lung Disease study.

**Methods:**

We analysed data from 4237 participants across 17 sites, mostly in LMICs. Occupational exposures were assessed by self-report (never vs ever) and the ALOHA+Job Exposure Matrix for vapours, gases, dusts, fumes (VGDF), pesticides, solvents and metals (cumulative exposure). Respiratory outcomes included: forced expiratory volume in 1 s (FEV₁), forced vital capacity (FVC), the FEV₁/FVC and respiratory symptoms. Associations were examined using multilevel linear and logistic regression models adjusted for age, sex, smoking, pack-years, education, body mass index and baseline FVC. We further explored sex differences and non-linear relationships for symptoms.

**Results:**

Over a median 10 years of follow-up, FEV₁/FVC decline was associated with moderate (β=–1.34; 95% CI –2.32 to –0.35) and high (β=–1.79; 95% CI –3.33 to –0.20) exposure to VGDF and low (β=–1.11; 95% CI –2.11 to –0.08) and high (β=–2.16; 95% CI –4.08 to –0.24) exposure to pesticides. Increased risk of wheeze was associated with moderate (Relative Risk (RR) =1.45; 95% CI 1.05 to 2.15) and high (RR=1.89; 95% CI 1.100 to 3.26) exposure to pesticides. Associations did not differ by sex. There was weak evidence of non-linear exposure–response relationships and no associations with solvents or metals.

**Conclusions:**

A significant decline in FEV_1_/FVC was associated with exposure to VGDF and pesticides. Increased risk of wheeze was also associated with exposure to pesticides. These findings underscore the need for continued monitoring in high-exposure settings, particularly in LMICs.

**WHAT IS ALREADY KNOWN ON THIS TOPIC**

- Respiratory symptoms and reduced lung function have been linked to occupational exposures, mainly from studies conducted in high-income countries. Longitudinal evidence from studies in low- and middle-income countries (LMICs) remains limited.

**What this study adds**

- Using longitudinal data from the multinational Burden of Obstructive Lung Disease cohort, this study shows that exposure to vapours, gases, dusts and fumes and pesticides is associated with small but significant decline in forced expiratory volume in 1 s/forced vital capacity over time as well as an increased risk for wheeze.

**How this study might affect research, practice or policy**

- By highlighting longitudinal respiratory effects in predominantly LMIC populations, this study reinforces the importance of occupational respiratory surveillance for early detection of lung function decline and respiratory symptoms. It also emphasises the need for routinely collecting occupational histories among workers in high-exposure settings, even before clinical disease develops.

## Introduction

In many settings, workers are frequently exposed to harmful agents, including vapours, gases, dusts, fumes (VGDF), solvents, metals and pesticides, which may impact their health. In low- and middle-income countries (LMICs), where limited regulatory oversight often results in higher occupational exposures, workers may experience a greater burden of respiratory disease compared with those in high-income countries (HICs), where occupational exposure limits are more consistently implemented and enforced.^1^

Several cross-sectional studies have reported associations between occupational exposures and reduced lung function in the general population,^2 3^ and while longitudinal studies have generally supported these associations, there is considerable heterogeneity in findings due to exposure assessment methods, study populations and respiratory outcomes examined.^4^ In addition, most of these studies have been conducted in HICs,^5 6^ limiting the generalisability of their findings to other world regions. Therefore, there is a critical need for longitudinal data from LMICs, where the burden and characteristics of occupational exposures may differ significantly due to differences in industrial practices, socioeconomic conditions and regulatory environments.^7 8^

The study by Ratanachina *et al*,^9^ using Burden of Obstructive Lung Disease (BOLD) data, showed significant cross-sectional associations between self-reported occupational exposures and respiratory symptoms, but not with lung function. Building on this, we aimed to investigate the longitudinal associations between occupational exposures and prospective risk of respiratory health outcomes using data from both the baseline and follow-up surveys of the BOLD cohort.

## Methods

### Study population

**Figure 1:**
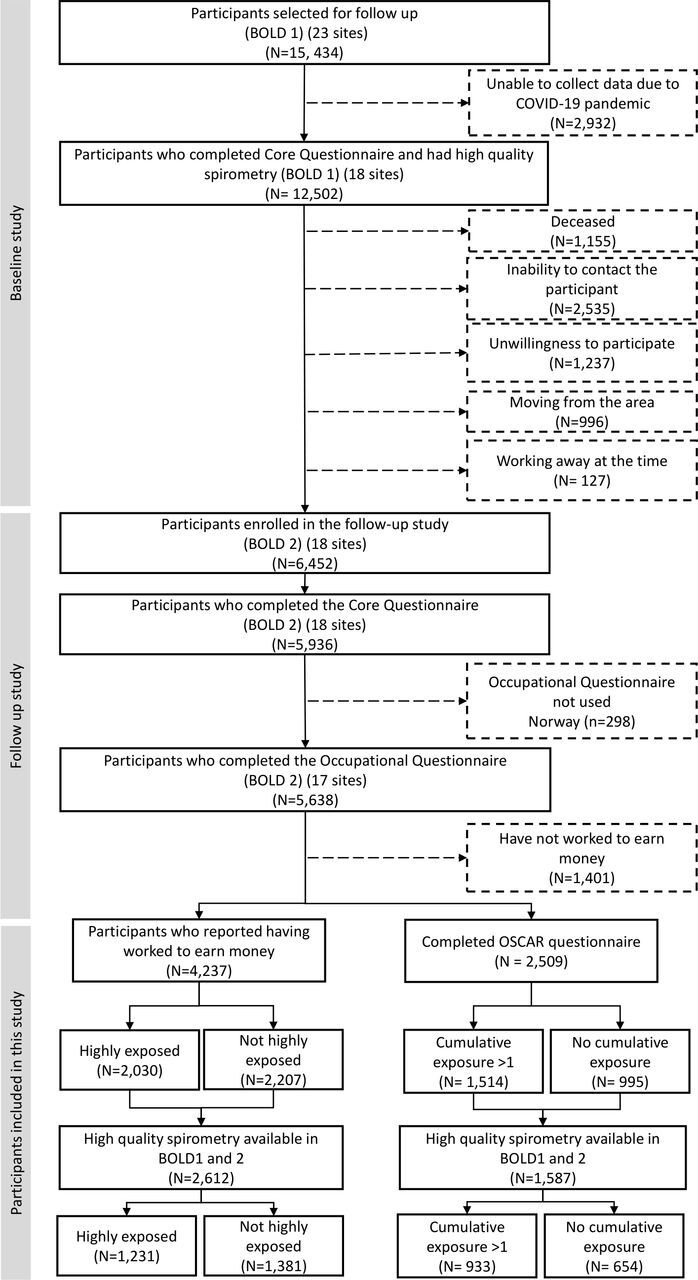
Flow chart of participants with occupational data available in BOLD 2. BOLD, Burden of Obstructive Lung Disease; OSCAR, Occupations Self-coding Automatic Recording.

In brief, the BOLD study was established to assess the prevalence of chronic airflow obstruction (CAO) and its main risk factors across the world.^10^ At baseline (2003–2016), adults aged 40 years and above were recruited from general populations in 41 sites across Africa, Asia, North America, Europe, the Caribbean and Australia. At follow-up (2019–2021), participants in 18 sites were seen again. Fourteen sites included in the follow-up study were in LMICs and four were in Northern Europe. On both occasions, study participants provided information on respiratory symptoms, medical diagnoses, and potential risk factors for chronic lung diseases. Additionally, they underwent physical measurements including spirometry. Of 12 502 eligible participants for follow-up, we excluded those who: had died (n=1155), could not be contacted (n=2535), were unwilling to participate (n=1237), had moved from the area (n=996) and were working away at the time (n=127) (figure 1). Of 6452 participants enrolled in the follow-up, 5936 completed the core questionnaire, and a total of 5638 also completed the occupational questionnaire. The occupational questionnaire was not used in the follow-up survey in Bergen (Norway) (figure 1). Questionnaires were translated and administered in local languages.

### Occupational exposures

Participants were asked whether they had ever worked for more than 3 months in one or more settings, selected a priori, as likely to be associated with high exposures to occupational agents. The occupations were grouped into exposure categories according to experts’ opinions and likelihood of exposure to at least one of (1) inorganic dusts; (2) organic dusts; (3) fumes and (4) cleaning products. The occupations included (1) farming; (2) flour, feed or grain milling; (3) cotton or jute processing; (4) hard-rock mining; (5) coal mining; (6) sandblasting; (7) working with asbestos; (8) chemical or plastics manufacturing; (9) foundry or steel milling; (10) welding; (11) firefighting; (12) construction; (13) cleaning; (14) cement manufacturing and (15) waste recycling. Participants who selected one or more of these settings were classified as ‘highly exposed’.

Participants were also asked to complete the Occupations Self-Coding Automatic Recording (OSCAR) questionnaire, which coded jobs held for longer than 3 months with an International Standard Classification of Occupations 88 (ISCO-88) code. OSCAR is a validated web-based tool developed to collect and code lifetime job histories in large population cohorts.^11^ These occupational histories were collected at follow-up and covered also jobs held before the baseline study. Occupational exposures were assigned using the ALOHA+Job Exposure Matrix (JEM).^12^ Each participant was assigned exposure to four main categories: (1) VGDF, (2) pesticides, (3) solvents and (4) metals. For each occupational agent, cumulative exposure was calculated by multiplying the duration of each job by the squared exposure intensity score assigned by the ALOHA+JEM (0, 1, 4), and summing these values across all reported jobs to derive cumulative exposure units (EU-years).^13^ These were categorised as never exposed, low (<median), moderate (≥median-to<90 th percentile) and high (≥90 th percentile) exposure.

### Lung function

Spirometry testing was conducted by trained technicians using a ndd EasyOne spirometer (ndd Medizintechnik AG, Zurich, Switzerland). Participants performed spirometry before and after the inhalation of 200mcg salbutamol completing a minimum of three acceptable manoeuvres. All lung function measurements were quality-controlled at the BOLD coordination centre based on American Thoracic Society guidelines. In our analyses, we used postbronchodilator measures. We defined CAO as a forced expiratory volume in 1 s/forced vital capacity (FEV_1_/FVC) less than the lower limit of normal (LLN)^14 15^ based on the third National Health and Nutrition Examination reference equations.^14^ We used these equations to make it easier to compare with previous BOLD study findings. Annual changes in FEV_1_ and FVC were calculated as the difference between follow-up measurements and baseline measurements, divided by the time between the two examinations.

### Respiratory symptoms

The presence of different respiratory symptoms was recorded at the time of the questionnaire. We defined wheeze as having had any whistling in the chest at any time in the last 12 months. Chronic cough as a frequent cough, without having a cold, on most days for at least 3 months each year. Chronic phlegm as a frequent production of phlegm, without a cold, on most days for at least 3 months each year. We defined dyspnoea using the modified Medical Research Council dyspnoea scale as breathlessness at least when walking more slowly than people of the same age or sufficient to have to stop walking.^9^

### Statistical analysis

We used mixed-effects linear regression models to assess the associations between occupational exposures and changes in FEV_1,_ FVC and FEV_1_/FVC. To assess the association between occupational exposures and incidence of CAO and respiratory symptoms, we used mixed-effects Poisson with robust error variance.^16^ Models were adjusted for age, sex, smoking status, smoking pack-years, educational level (primary: no education or primary level, secondary: middle school/high school level, tertiary: technical/vocational college/university), body mass index (BMI; kg/m²) and baseline FVC or respiratory symptoms. Study site was entered in the models as random intercept. We excluded participants with CAO (FEV_1_/FVC<LLN) at baseline when assessing CAO incidence at follow-up and used inverse probability weights to account for loss to follow-up in all of our analyses. We also conducted analyses in the ‘highly exposed’ subpopulation stratified by sex and smoking status. We tested for interactions by entering interaction terms between occupational exposure and sex or smoking status. However, we could not do this using ALOHA+JEM exposure estimates due to smaller sample sizes within subgroup strata.

We performed an additional analysis using restricted cubic splines, with knots placed at the 10th, 50th and 90th percentiles of the agent exposure distribution (EU-years) to investigate non-linear dose-response relationships between occupational exposures and respiratory symptoms.

All analyses were conducted using R programming language and R Studio using the *lme4* and *rms* packages.^17^

### Sensitivity analyses

First, we restricted the sample to participants from the 14 LMIC sites and re-ran all the models above. This was conducted in those who completed the occupational questionnaire on high-risk occupations and OSCAR questionnaire independently. A corresponding HIC-only analysis was not feasible because only three HIC sites contributed data, below the minimum number of clusters required for stable estimation of random effects. Second, to assess the potential influence of variable response rates across the sites, we repeated all analyses in the subset of countries with a response rate >50%.

## Results

Over a median follow-up time of 10.1 years (IQR: 6.3–11.0), 4237 participants completed the occupational questionnaire on highly exposed settings, and 2509 completed the occupational OSCAR questionnaire (figure 1 and online supplemental table 1). Across all 17 sites, the highest response rate was in Iceland (Reykjavik) with 99.5% and the lowest was in Pakistan (Karachi) with 44.4% (online supplemental figure 1). The response rate to the OSCAR questionnaire ranged from 13.3% in India (Mysore) to 86.0% in Iceland (Reykjavik).

10.1136/bmjresp-2026-004213.supp1 Supplementary data

### Participants in ‘highly exposed’ occupational settings

From the 4237 participants that reported paid work in the past, a total of 2030 (47.9%) were categorised ‘highly exposed’ (figure 1). The highest proportion of participants likely exposed to inorganic dust was in Morocco (Fes, 84.6%), while workers likely exposed to organic dusts were more common in rural India (Pune, 95.5%). Workers likely exposed to fumes were more common in Sweden (Uppsala, 50.6%) and most likely exposed to cleaning products were in Iceland (Reykjavik, 35.9%) (online supplemental table 2).

Over half participants in the ‘highly exposed’ group were males (59.7%), with a mean age of 62 years at follow-up, 38.8% completed education up to secondary level and 61.6% were never smokers. The median employment duration was 21 years (IQR 8–38). We observed lung function decline over time with mean FEV_1_ and FVC decreasing by 26.8 mL/year and 27.1 mL/year, respectively. The prevalence of CAO increased from 7.5% to 8.5%. The FEV₁/FVC ratio declined slightly from 78.5% to 77.0% (–0.17%). Among participants who completed the respiratory symptoms questionnaire, wheezing increased from 12.4% to 13.5% and dyspnoea from 9.2% to 11.6%. However, chronic cough decreased from 7.5% to 6.0%, and chronic phlegm from 6.2% to 5.7% (table 1).

**Table 1:** Characteristics of participants with occupational data and high-quality spirometry data available at the Burden of Obstructive Lung Disease baseline and follow-up surveys

	Completed occupational questionnaire on highly exposed settings (N=2612)		Completed OSCAR questionnaire (N=1587)	
Characteristic	Baseline	Follow-up	Baseline	Follow-up
Sex, females, n (%)	1249 (47.8)	1249 (47.8)	757 (47.7)	757 (47.7)
Age, years, mean (SD)	52 (9.2)	61.4 (9.8)	50.9 (8.4)	60.7 (9.1)
Body mass index (kg/m^2^), mean (SD)	25.7 (5.4)	26.8 (7.4)	25.5 (5.3)	26.9 (7.8)
Never smokers, n (%)	1823 (69.7)	1744 (67.9)	1066 (67.1)	1045 (65.8)
Ever smokers, n (%)	789 (30.2)	826 (31.6)	521 (32.8)	528 (33.4)
Smoking pack years, median (IQR)	14.3 (5.0–27.0)	13.5 (4.7–32.0)	14.5 (5.0–26.2)	13.5 (5.0–31.0)
Follow-up time, years, median (IQR)	–	10.1 (6.3–11.0)	–	9.8 (8.1–11.2)
Duration of employment, years, median (IQR)	21 (8–38)			30 (15–40)
Education level, N (%)				
None to primary	–	814 (31.2)	–	496 (31.3)
Secondary	–	970 (37.1)	–	581 (36.6)
Tertiary	–	828 (31.7)	–	510 (32.1)
FVC, mean (SD), L	3.3 (0.9)	3.0 (0.9)	3.4 (1.0)	3.1 (0.9)
FEV_1_, mean (SD), L	2.6 (0.8)	2.3 (0.7)	2.7 (0.8)	2.4 (0.7)
FEV_1_/FVC %, mean (SD)	78.5 (7.6)	77.0 (7.7)	79.3 (6.9)	77.3 (7.3)
CAO, n (%)	195 (7.5)	221 (8.5)	97 (6.1)	118 (7.4)
*Respiratory symptoms, n (%)				
Wheezing^a^	495 (12.4)	575 (13.5)	279 (12.0)	342 (13.6)
Chronic cough^b^	299 (7.5)	244 (6.0)	146 (6.2)	144 (5.9)
Chronic phlegm^c^	209 (6.2)	217 (5.7)	100 (4.3)	124 (5.1)
Dyspnoea^a^	366 (9.2)	396 (11.6)	158 (6.6)	209 (8.6)

*Respiratory symptoms were derived from self-reported questionnaires, independent of lung function testing. Therefore, the N for symptoms is as follows: Completed occupational questionnaire on highly exposed settings:

Completed occupational questionnaire on highly exposed settings: ^a^ Wheezing and dyspnoea N=3929; ^b^ Chronic cough N=3771; ^c^ Chronic phlegm N=3774.

Completed OSCAR questionnaire: ^a^ Wheezing N=2509; ^b^ Chronic cough and chronic phlegm N=2423; ^c^ Dyspnoea N=2096.

CAO, chronic airflow obstruction; FEV1, forced expiratory volume in 1 s; FVC, forced vital capacity; OSCAR, Occupations Self-coding Automatic Recording.

Overall, participants working in ‘highly exposed’ occupations did not show a significant decline in FEV₁/FVC (β=−0.45, 95% CI −0.99 to 0.08). The association between highly exposed occupations and FEV₁/FVC appeared stronger among males (β=−0.83, 95% CI −1.60 to −0.05) than females (β=0.02, 95% CI −0.73 to 0.76); however, interaction tests did not support statistically significant effect modification by sex. No associations were found between highly exposed occupations and FEV_1_, FVC or the risk of CAO at follow-up (table 2).

**Table 2:** Associations between ‘highly exposed’ occupations and respiratory outcomes over time*

Outcome	Overall (n/N)	Overall β (95% CI)	Males (n/N)	Male β (95% CI)	Females (n/N)	Females β (95% CI)	P-int sex	Never smokers (n/N)	Never smokers β (95% CI)	Ever smokers (n/N)	Ever smokers β (95% CI)	P-int smoking
FEV₁/FVC	1231/2612	−0.45 (−0.99 to 0.08)	728/1347	−0.83 (−1.60 to −0.05)	503/1265	0.02 (−0.73 to 0.76)	0.096	798/1744	−0.36 (−1.03 to 0.31)	422/826	−0.74 (−1.65 to 0.17)	0.089
FEV₁	1231/2612	−0.01 (−0.05 to 0.03)	728/1347	−0.06 (−0.07 to 0.06)	503/1265	−0.02 (−0.07 to 0.03)	0.085	798/1744	−0.03 (−0.08 to 0.02)	422/826	0.03 (−0.04 to 0.09)	0.078
FVC	1231/2612	0.02 (−0.04 to 0.05)	728/1347	0.03 (−0.04 to 0.10)	503/1265	−0.03 (−0.09 to 0.04)	0.087	798/1744	−0.02 (−0.08 to 0.03)	422/826	0.06 (−0.05 to 0.14)	0.083
CAO	221/2612	0.97 (0.68 to 1.38)	148/1347	0.77 (0.51 to 1.16)	73/1265	1.51 (0.70 to 3.27)	0.550	101/1744	1.18 (0.67 to 2.07)	120/826	0.98 (0.60 to 1.56)	0.375
	Overall (n/N)	Overall RR (95% CI)	Males (n/N)	Male RR (95% CI)	Females (n/N)	Females RR (95% CI)	P-int sex	Never smokers (n/N)	Never smokers RR (95% CI)	Ever smokers (n/N)	Ever smokers RR (95% CI)	P-int smoking
Dyspnoea	209/3929	1.12 (0.78 to 1.65)	90/1905	0.98 (0.72 to 1.33)	119/2024	1.30 (0.81 to 2.08)	0.284	155/2500	1.30 (0.81 to 2.10)	54/1276	0.93 (0.46 to 1.85)	0.242
Wheeze	266/3929	1.29 (1.03 to 1.60)	146/1905	1.23 (0.93 to 1.64)	120/2024	1.43 (1.05 to 1.96)	0.386	131/2500	1.42 (1.02 to 1.97)	135/1276	1.25 (0.91 to 1.67)	0.387
Chronic cough	208/3771	1.06 (0.76 to 1.50)	102/1960	1.30 (0.79 to 2.10)	106/1811	0.96 (0.59 to 1.56)	0.332	109/2499	1.05 (0.65 to 1.70)	99/1272	1.25 (0.76 to 2.06)	0.328
Chronic phlegm	181/3774	1.05 (0.74 to 1.49)	102/1961	1.10 (0.68 to 1.78)	79/1813	0.97 (0.55 to 1.72)	0.367	87/2500	0.76 (0.44 to 1.31)	74/1274	1.45 (0.90 to 2.33)	0.373

CAO, chronic airflow obstruction; FEV_1_, forced expiratory volume in 1 s; FVC, forced vital capacity; P-int, p value for the interaction; RR, Relative risk.

Wheeze was associated with ‘highly exposed’ occupations (RR=1.29, 95% CI 1.03 to 1.60). Associations with wheeze appeared somewhat stronger among females (RR=1.43, 95% CI 1.05 to 1.96) and never smokers (RR=1.42, 95% CI 1.02 to 1.97), although no statistically significant interactions by sex or smoking status were observed. No evidence of associations was found with chronic cough, chronic phlegm or dyspnoea in this subpopulation (table 2).

### Participants with completed OSCAR questionnaire

Among the 2509 participants that completed the OSCAR questionnaire, the most common job titles were crop farm labourers (N=444), retail and wholesale trade (N=198) and mixed crop and animal producers (N=156). Over half had exposure to at least one occupational agent of interest (60.3%). The most common exposure was VGDF (N=1512), followed by pesticides (N=683), solvents (N=484) and metals (N=168). Over half were males (60.9%), with a mean age of 60.4 years at follow-up, 60% were never smokers and 39.2% completed education up to secondary level. The median duration of employment was 30 years (IQR 15–40) (online supplemental table 1). Among these, the mean FEV₁ and FVC declined by 25.3 mL/year and 25.1 mL/year, respectively. The prevalence of CAO increased from 6.1% to 7.4%. Respiratory symptoms became more common: wheeze rose from 12.0% to 13.6%, chronic phlegm from 4.3% to 5.1% and dyspnoea from 9.2% to 11.6%. In contrast, the prevalence of chronic cough decreased from 6.2% to 5.9% (table 1).

Among participants that completed the OSCAR questionnaire, reduction in FEV₁/FVC was associated with moderate (β=−1.34, 95% CI −2.32 to −0.35) and high exposure to VGDF (β=−1.79, 95% CI −3.33 to −0.20). Reduction in FEV₁/FVC was also associated with low (β=−1.11, 95% CI −2.11 to −0.08) and high pesticide exposure (β=−2.16, 95% CI −4.08 to −0.24). CAO, FEV_1_ and FVC were not associated with occupational exposures in this subpopulation (table 3, online supplemental figure 2).

**Table 3:** Associations between occupational exposures (ALOHA+JEM) and lung function outcomes over time

	FEV_1_/FVC		FEV_1_		FVC		FEV_1_/FVC<LLN	
ALOHA+JEM agent	N	β (95% CI)	N	β (95% CI)	N	β (95% CI)	N	RR (95% CI)
VGDF								
Low (<68 EU-years)	442	−0.23 (−1.02 to 0.56)	442	−0.01 (−0.04 to 0.06)	442	0.01 (−0.07 to 0.06)	33	1.34 (0.76 to 2.45)
Moderate (68–155 EU-years)	404	−1.34 (−2.32 to −0.35)	404	−0.02 (−0.09 to 0.04)	404	0.01 (−0.07 to 0.09)	38	1.61 (0.88 to 2.94)
High (≥156 EU years)	85	−1.79 (−3.33 to −0.20)	85	−0.03 (−0.14 to 0.06)	85	−0.01 (−0.13 to 0.10)	11	1.27 (0.54 to 2.98)
All pesticides								
Low (≤147 EU-years)	212	−1.11 (−2.11 to −0.08)	212	−0.02 (−0.09 to 0.06)	212	0.01 (−0.09 to 0.08)	17	0.73 (0.35 to 1.49)
Moderate (148–179 EU-years)	177	−1.16 (−2.39 to 0.07)	177	−0.01 (−0.09 to 0.09)	177	0.03 (−0.09 to 0.14)	16	1.04 (0.51 to 2.12)
High (≥180 EU-years)	85	−2.16 (−4.08 to −0.24)	85	−0.02 (−0.11 to 0.10)	85	0.09 (−0.06 to 0.25)	7	0.97 (0.39 to 2.40)
All solvents				–				
Low (<38 EU-years)	135	−0.38 (−1.52 to 0.77)	135	−0.01 (−0.08 to 0.07)	135	−0.01 (−0.10 to 0.08)	12	1.20 (0.61 to 2.35)
Moderate (38–45 EU-years)	119	−0.57 (−1.83 to 0.69)	119	−0.04 (−0.12 to 0.04)	119	−0.04 (−0.14 to 0.07)	8	0.95 (0.46 to 1.96)
High (≥46 EU-years)	45	−0.84 (−2.34 to 1.47)	45	−0.02 (−0.14 to 0.11)	45	−0.01 (−0.06 to 0.15)	6	–
Metals								
Low (<31 EU-years)	51	−0.28 (−1.87 to 1.15)	51	0.07 (−0.05 to 0.18)	51	0.11 (−0.03 to 0.25)	5	–
Moderate (31–44 EU-years)	41	−1.55 (−3.34 to 0.31)	41	−0.02 (−0.15 to 0.11)	41	0.04 (−0.12 to 0.20)	5	–
High (≥45 EU-years)	12	−1.39 (−4.53 to 1.76)	12	−0.03 (−0.26 to 0.20)	12	0.06 (−0.22 to 0.35)	1	–

EU-years, cumulative exposure units calculated from exposure intensity×duration of employment; FEV_1_, forced expiratory volume in 1 s; FVC, forced vital capacity; JEM, Job exposure matrix; LLN, lower limit of normal; RR, Relative risk; VGDF, vapours, gases, dusts, fumes.

The risk of wheeze was increased for moderate (Relative Risk (RR)=1.89, 95% CI 1.10 to 3.26) and high (RR=1.45, 95% CI 1.02 to 2.15) pesticide exposure. Chronic phlegm was less likely to be reported by those with low (RR=0.60, 95% CI 0.45 to 0.97) and moderate VGDF exposure (RR=0.50, 95% CI 0.36 to 0.98). We found no associations between other occupational exposures and chronic cough or dyspnoea. Although many estimates were not statistically significant, the direction of effect consistently suggested a decline in lung function and/or an increased risk to respiratory symptoms, particularly in the highest exposure categories (table 4, online supplemental figure 3).

**Table 4:** Associations between occupational exposures (ALOHA+JEM) and respiratory symptoms over time

	Wheeze		Chronic cough		Chronic phlegm		Dyspnoea	
	N	RR (95% CI)	N	RR (95% CI)	N	RR (95% CI)	N	RR (95% CI)
VGDF								
Low (<68 EU-years)	99	1.01 (0.76 to 1.34)	47	1.11 (0.73 to 1.66)	29	0.60 (0.45 to 0.97)	45	0.90 (0.47 to 1.71)
Moderate (68–155 EU-years)	71	1.14 (0.80 to 1.61)	19	0.70 (0.37 to 1.29)	16	0.50 (0.36 to 0.98)	54	0.92 (0.37 to 2.37)
High (≥156 EU years)	19	1.27 (0.71 to 2.25)	6	1.02 (0.39 to 2.60)	5	–	28	0.99 (0.22 to 4.41)
All pesticides								
Low (≤147 EU-years)	36	1.13 (0.85 to 2.20)	5	0.83 (0.37 to 1.83)	7	0.77 (0.44 to 1.37)	25	1.76 (0.76 to 4.05)
Moderate (148–179 EU-years)	28	1.45 (1.05 to 2.15)	3	–	5	1.19 (0.55 to 2.57)	33	2.38 (0.90 to 6.34)
High (≥180 EU-years)	15	1.89 (1.10 to 3.26)	5	1.31 (0.56 to 3.03)	3	–	23	1.90 (0.52 to 6.90)
All solvents								
Low (<38 EU-years)	33	1.01 (0.66 to 1.52)	10	0.82 (0.47 to 1.43)	11	0.83 (0.53 to 1.30)	12	1.07 (0.43 to 2.34)
Moderate (38–45 EU-years)	36	1.13 (0.76 to 1.66)	13	0.81 (0.52 to 1.23)	11	1.04 (0.53 to 2.03)	7	0.69 (0.21 to 1.90)
High (≥46 EU-years)	9	0.93 (0.48 to 1.78)	4	1.21 (0.56 to 2.50)	1	–	4	–
Metals								
Low (<31 EU-years)	12	1.02 (0.52 to 1.97)	6	1.45 (0.78 to 2.71)	5	1.04 (0.44 to 2.65)	3	–
Moderate (31–44 EU-years)	11	1.11 (0.60 to 2.04)	5	1.17 (0.43 to 1.66)	4	–	2	–
High (≥45 EU-years)	4	1.32 (0.45 to 3.87)	2	–	0	–	1	–

EU-years, cumulative exposure units calculated from exposure intensity × duration of employment; JEM, Job Exposure Matrix; RR, Relative risk; VGDF, vapours, gases, dusts, fumes.

No statistically significant non-linear associations were observed (online supplemental figure 4 through figure 7).

### Sensitivity analyses

When excluding participants from European (HIC) sites, participants working in highly exposed occupations in LMIC settings showed a statistically significant decline in FEV₁/FVC (β = –0.68, 95% CI –1.33 to –0.03) with larger effect estimates observed in analyses restricted to LMIC sites compared with analyses including both LMIC and HIC sites. The overall risk of wheeze also remained statistically significant (RR=1.44, 95% CI 1.09 to 1.92), with higher risks observed among females (RR=1.85, 95% CI 1.27 to 2.69) and never smokers (RR=1.52, 95% CI 1.02 to 2.29). No associations were identified between highly exposed occupations and FEV₁, FVC, or the risk of CAO, chronic cough, chronic phlegm or dyspnoea at follow-up (online supplemental table 3).

A significant FEV_1_/FVC decline was observed among participants with moderate (β=−1.98, 95% CI −3.20 to −0.76) and high (β=−2.91, 95% CI −4.79 to −1.04) VGDF exposure. Similar results were observed with low (β=−1.44, 95% CI −2.69 to −0.19), moderate (β=−1.48, 95% CI −2.95 to −0.02) and high (β=−2.99, 95% CI −5.12 to −0.86) pesticide exposure. Overall, the effect estimates were larger than those observed in analyses including all sites. There was also an increased risk of wheeze among participants with moderate (RR=1.54, 95% CI 1.05 to 2.15) and high (RR=2.20, 95% CI 1.17 to 4.14) pesticide exposure. There were no other statistically significant associations (online supplemental table 4).

After restricting the analysis to sites with an OSCAR questionnaire response rate >50% (Benin (Sèmè-Kpodji), Estonia (Tartu), Iceland (Reykjavik), India (Pune), Nigeria (Ile-Ife), Sudan (Khartoum), Sweden (Uppsala); N=1093), there was an increased risk of CAO at follow-up (RR=2.20, 95% CI 1.07 to 3.74) in participants with moderate exposure to VGDF. The risk of wheeze was also increased in participants with moderate (RR=1.70, 95% CI 1.01, 2.86) and high (RR=2.16, 95% CI 1.11 to 4.22) exposure to pesticides. The risk of dyspnoea was also higher in participants with moderate (RR=2.22, 95% CI 1.53 to 6.34) exposure to pesticides. There were no statistically significant associations observed for FEV_1_/FVC, FEV_1_, FVC, chronic cough or chronic phlegm across any exposure category (online supplemental table 5).

## Discussion

In this multinational study, we identified associations between occupational exposures and adverse respiratory outcomes across diverse LMIC populations. We compared two approaches to exposure assessment: self-reported occupational history using the core questionnaire and using the ALOHA+JEM (those with completed OSCAR questionnaire). Both approaches showed consistently that exposures to VGDF and pesticides were associated with small but statistically significant reductions in lung function and increased risk of respiratory symptoms over time.

The decline in FEV₁/FVC observed over a median follow-up of 10 years among participants with occupational exposures was modest at the individual level but may still be relevant at the population level, particularly in settings with sustained or poorly controlled exposures. Previous longitudinal general population studies of lung function decline showed differences ranging from small associations with occupational agents^5 12 18^ to no associations.^19^ When exposures were assigned using the ALOHA+JEM, we also observed significant reductions in FEV₁/FVC, associated with both moderate and high VGDF exposure, and with low and high levels of pesticide exposure, suggesting a dose-response relationship. Additionally, the risk of wheeze was significantly higher among participants with moderate and high pesticide exposure. While no statistically significant associations were observed between JEM-based exposures and CAO, or respiratory symptoms, the overall direction of effect, that is, increased exposure associated with declining lung function and increased symptom risk, remained consistent.

We observed an unexpected negative association between VGDF exposure and chronic phlegm. However, chronic phlegm is a non-specific symptom that can be caused by a wide range of non-respiratory conditions such as sinus disease, gastro-oesophageal reflux, and heart failure, which may in part explain this finding.^20^ Another possibility is the healthy worker effect, where individuals in physically demanding jobs tend to be healthier overall, as those with health issues may avoid or leave such work.^21^ It may also be that individuals with chronic phlegm reduce their exposure or leave high-exposure jobs. In addition, as participants were asked about exposure up to baseline, it is possible that much of the exposure occurred in the more distant past, and symptoms like chronic phlegm may have since resolved, leading to an apparent lack of association. Finally, the protective effect may be artefactual, driven by small sample sizes in higher exposure categories or unaccounted heterogeneity within groups.

To address the limitations of stratified analyses and better capture potential non-linear dose–response relationships, we modelled occupational exposures as continuous variables using restricted cubic splines. While we only observed a borderline significant non-linear association between solvent exposure and chronic cough, these analyses suggested varying patterns of association between exposure levels and respiratory outcomes, with differences across exposure agents and symptom types. Both the categorical and continuous exposure models consistently identified increased risks of wheeze associated with pesticide and VGDF exposures, particularly at higher levels. These findings may reflect asthma-like pathophysiology. A previous study of agricultural workers in India found that occupational exposure to organophosphate and carbamate pesticides, which inhibit acetylcholinesterase, can lead to mucus hypersecretion, airway inflammation, bronchospasm, breathlessness and wheeze over time.^22^ These mechanisms support the plausibility that chronic low-level pesticide exposure may contribute to airway hyperreactivity and obstructive respiratory changes, even in the absence of clinically diagnosed asthma.^23^ However, the reliability of spline estimates at high exposure levels is limited due to small sample size, and these results should be interpreted with caution.

A previous analysis using only BOLD baseline data reported associations between self-reported occupational exposures and respiratory symptoms, but not with reductions in lung function (FEV₁/FVC or FVC).^9^ In the current analysis, we used longitudinal data, which may explain the emergence of new associations.^9^ The analytic sample was smaller in this analysis, as it was limited to participants with both spirometry and occupational history data, potentially introducing selection bias. Additionally, lower life expectancy in LMICs, particularly among individuals with higher disease burden or reduced access to healthcare, may have led to early mortality and underrepresentation of severe cases at follow-up.^24 25^ However, restricting analyses to LMIC sites highlighted stronger and consistent associations with FEV_1_/FVC decline in participants exposed to occupational VGDF and pesticides. This reinforces the main findings suggesting that occupational exposures may have greater respiratory impact in LMIC environments. This is likely due to higher and persistent exposure levels or weaker protective regulations. Because OSCAR response rates varied widely across sites, we repeated the analyses in countries with a response rate >50%. In this higher-response subset, most lung function associations remained null, but elevated risks of CAO with moderate VGDF exposure and increased wheeze and dyspnoea with moderate or high pesticide exposure were still observed. Although precision was limited by smaller sample sizes, these findings support the robustness of the main associations of respiratory symptoms with exposure to VGDF and pesticides.

This study has strengths, including the large sample size and a diverse population, predominantly from LMICs. We could also compare the findings when assigning exposures using both self-reported data and a semiquantitative JEM. However, there are limitations. Self-reported symptoms are subject to recall bias and cultural differences in perception and reporting, which in turn result in lower prevalences and limited statistical power. We only had one additional spirometry measurement at follow-up, the observed differences in lung function over time may be explained by intra-individual variability. Additionally, it may be too short to observe chronic health effects resulting from some occupational exposures. Moreover, the varying follow-up periods across sites may not be long enough for people to fall below the LLN thresholds to develop detectable obstruction, particularly if starting with a high value. The significant loss to follow-up due to COVID-19 pandemic may have contributed to the variability in questionnaire response rates introducing selection bias, although we used inverse probability weighting to account for this. In analyses of incident CAO, excluding participants with CAO at baseline may have underestimated associations if occupational exposures had already contributed to disease before study enrolment. The ALOHA+JEM may not capture individual variability in exposure intensity or job title. In the analyses we did not account for co-exposures. However, our previous study showed that adjusting for co-exposures had little impact on results, except for VGDF.^2^ Furthermore, the prevalence of informal jobs is much higher in LMICs, and accurate job coding is very challenging; these jobs may not have been reflected in the JEM, resulting in underestimation of exposures. Furthermore, the ALOHA+JEM is largely based on data from HIC and is less applicable to the LMIC setting.^26^ In addition, the exposure classification relies on ISCO-88 coding, and it remains unclear whether this classification fully reflects contemporary occupational structures in LMICs or whether more recent coding systems might have improved exposure assignment. Although the ALOHA+JEM was not developed or validated specifically for LMIC settings, it remains a pragmatic and structured approach in settings where detailed exposure measurements are unavailable, such as in large cohort studies, offering advantages over reliance on self-reported data alone. Some imprecise estimates in the spline analyses may stem from exposure misclassification (eg, reliance on JEMs rather than exposure monitoring), unmeasured confounding (eg, use of protective equipment, ambient pollution), or limited outcome prevalence in exposure strata. Nevertheless, the direction of point estimates aligns with previous population-based studies, for instance occupational exposure to metals and VGDFs are known as independent risk factors for lung function decline.^6^ Future longitudinal occupational studies should identify specific agents that increase the risk of chronic respiratory diseases, facilitating targeted exposure controls. Additionally, there is need for more epidemiological research on occupational lung diseases in LMICs, where regulatory oversight may be weaker and exposure burdens higher. Experimental studies exploring novel mechanisms (eg, DNA methylation or inflammatory pathways) linked to early manifestations of lung disease are warranted. Early diagnosis remains essential to prevent further lung function decline, thereby reducing both health and socio-economic burdens. Future initiatives should emphasise taking a structured occupational history. While occupational health surveillance is often limited in LMICs, preventive strategies such as exposure reduction, worker education and basic health monitoring represent practical starting points for creating safer and healthier workplaces.

## Conclusions

This longitudinal study confirms previous reports of association between occupational pesticide exposure and wheeze. It also shows that occupational exposures to VGDF and pesticides may lead to lung function decline. Although the observed effect sizes were modest at the individual level, these findings underscore the importance of respiratory monitoring in affected workers, particularly in less regulated settings.

## Data Availability

Data are available on reasonable request. The study data are not freely accessible. However, proposals for collaboration will be considered. Requests should be directed to the project lead, Dr Andre F S Amaral, a.amaral@imperial.ac.uk.
