# Supplementary material for: Association of respiratory health with occupational exposures in the Burden of Obstructive Lung Disease (BOLD) cohort: a multinational longitudinal study
Source: BMJ Open Respir Res. 2026 Jul 24;13(1):e004213. doi: 10.1136/bmjresp-2026-004213 (PMC13410711; doi:10.1136/bmjresp-2026-004213)

**Association of respiratory health with occupational exposures in the Burden of Obstructive Lung Disease (BOLD) cohort: A multinational longitudinal study**

**Valentina Quintero-Santofimio[1], Jixuan Ma[1,2], James Potts[1], Hans Kromhout[3], Johanna Feary[1,4], Christer Janson[5], Magnus Svartengren[6], Mahesh Padukudru Anand[7], Rain Jõgi[8], Thorarinn Gíslason[9,10], Sanjay Kamlakar Juvekar[11,12], Rune Nielsen[13,14], Gregory Erhabor[15], Imed Harrabi[16], Graham Dervereux[17], Andrei Malinovschi[18], Dhiraj Agarwal[19], Rana Ahmed[20,21], Vanessa Garcia-Larsen[22], Asaad Nafees[23], Peter Burney[1,] Andre Amaral[1,4], on behalf of the BOLD Collaborative Research Group‡**

1. National Heart and Lung Institute, Imperial College London, London, UK
2. Department of Occupational and Environmental Health, School of Public Health, Tongji Medical College, Huazhong University of Science and Technology, Wuhan, Hubei China.
3. Institute for Risk Assessment Sciences, University of Utrecht, Utrecht, The Netherlands
4. NIHR Imperial Biomedical Research Centre, London, UK
5. Department of Medical Sciences, Respiratory, Allergy and Sleep Research, Uppsala university, Uppsala, Sweden
6. Department of Medical Sciences, Department of Medical Sciences, Occupational and Environmental Medicine, Uppsala university hospital, Uppsala, Sweden, Uppsala university, Uppsala, Sweden
7. Department of Respiratory Medicine, JSS Medical College, JSSAHER, Mysore, Karnataka, India
8. Lung Clinic, Tartu University Hospital, Tartu, Estonia
9. Faculty of Medicine, University of Iceland, Reykjavik, Iceland.
10. Department of Sleep, Landspitali - The National University Hospital of Iceland, Reykjavik, Iceland
11. Vadu Rural Health Program, KEM Hospital Research Centre, Pune, India
12. Dr. D.Y. Patil Medical College, Hospital and Research Centre, Dr. D.Y. Patil Vidyapeeth, Pimpri, Pune, India
13. Department of Clinical Medicine, University of Bergen, Bergen, Norway
14. Department of Thoracic Medicine, Haukeland University Hospital, Bergen, Norway
15. Department of Medicine, Obafemi Awolowo University/Obafemi Awolowo University Teaching Hospitals Complex, Osun, Nigeria
16. Ibn El Jazzar Faculty of Medicine of Sousse, University of Sousse, Sousse, Tunisia
17. Clinical Sciences, Liverpool School of Tropical Medicine, Liverpool, UK
18. Department of Medical Sciences, Clinical Physiology, Uppsala University, Uppsala, Sweden
19. Vadu Rural Health Program, KEM Hospital Research Centre, Pune, India
20. The Epidemiological Laboratory (Epi-Lab), Khartoum, Sudan
21. Centre for Chronic Disease and Population Health Research, School of Population Health, RCSI University of Medicine and Health Sciences, Dublin, Ireland
22. Department of International Health, Bloomberg School of Public Health, Johns Hopkins University
23. Department of Community Health Sciences, Aga Khan University, Karachi, Pakistan

**Corresponding author:** Valentina Quintero Santofimio, National Heart and Lung Institute, Imperial College London, London, UK, vq20@imperial.ac.uk

**Supplementary Tables and Figures**

**Table S1.** Characteristics of Burden of Obstructive Lung Disease (BOLD) participants that completed the core occupational questionnaire and the OSCAR questionnaire categorised by exposure to occupational agents.

| | **Core occupational questionnaire** | | **OSCAR questionnaire** | |
|---|---|---|---|---|
| **Characteristic** | **Participants with highly exposed occupations (N=2,030)** | **Participants with not highly exposed occupations (N=2,207)** | **Participants with cumulative exposure >1 to any agent (N=1,514)** | **Participants without cumulative exposure (N=995)** |
| **Sex, N (%)** | | | | |
| Males | 1,212 (59.7) | 1,010 (45.7) | 922 (60.9) | 423 (42.5) |
| Age at follow up, mean (SD) | 62.3 (9.9) | 61.7 (10.4) | 60.4 (8.8) | 61.4 (9.6) |
| Duration of employment, years, median (IQR) | 21 (8-38) | N/A | 30 (15-40) | 25 (14-35) |
| **Education level, N (%)** | | | | |
| None to primary | 768 (37.8) | 437 (19.8) | 536 (35.4) | 194 (19.5) |
| Secondary | 789 (38.8) | 833 (37.8) | 593 (39.2) | 330 (33.2) |
| Tertiary | 462 (22.7) | 931 (42.2) | 385 (25.4) | 471 (47.3) |
| Body mass index ($kg/m^2$), mean (SD) | 26.0 (6.0) | 27.6 (8.5) | 26.3 (8.9) | 27.7 (5.9) |
| **Smoking status, N (%)** | | | | |
| Never | 1,207 (61.6%) | 1,428 (67.5%) | 897 (59.2) | 613 (61.7) |
| Current | 220 (11.2%) | 174 (8.2%) | 200 (13.3) | 76 (7.7) |
| Previous | 533 (27.2%) | 512 (24.2%) | 371 (24.5) | 269 (27.1) |
| Smoking pack years, median (IQR) | 14.7 (4.8-36.0) | 12.4 (4.5-22.0) | 16.0 (6.0-36.7) | 11.0 (3.8-24.7) |
| **Lung function** | | | | |
| | **Participants with highly exposed occupations (N=1,231)** | **Participants with not highly exposed occupations (N=1,381)** | **Participants with cumulative exposure >1 to any agent (N=933)** | **Participants without cumulative exposure (N=654)** |
| Post-bronchodilator FVC (L), mean (SD) | 3.1 (1.0) | 3.1 (0.9) | 3.2 (0.9) | 3.2 (0.9) |
| Post-bronchodilator $FEV_1$ (L), mean (SD) | 2.3 (0.8) | 2.4 (0.7) | 2.4 (0.7) | 2.4 (0.7) |
| Post-bronchodilator $FEV_1$/FVC, mean (SD) | 76.4 (8.3) | 77.7 (7.1) | 77.1 (7.6) | 78. 1 (6.8) |
| $FEV_1$/FVC< LLN (%) | 125 (10.1) | 96 (6.9) | 84 (9.0) | 34 (5.1) |
| **Respiratory symptoms, n (%)** | | | | |

| | | | | |
|---|---|---|---|---|
| Dyspnoea [a] | 242 (11.9) | 154 (7.0) | 142 (11.3) | 67 (7.9) |
| Chronic cough [b] | 117 (5.9) | 127 (6.1) | 79 (5.3) | 65 (6.7) |
| Chronic phlegm [c] | 111 (5.6) | 106 (5.0) | 60 (4.1) | 64 (6.7) |
| Wheeze | 318 (15.6) | 257 (11.6) | 212 (14) | 130 (13.0) |

[a] Cumulative exposure >1 N= 1,251; no cumulative exposure N= 845

[b] Cumulative exposure >1 N= 1,466; no cumulative exposure N= 957;
Highly exposed N= 1,957, not highly exposed N= 2,112

[c] Cumulative exposure >1 N= 1,466; no cumulative exposure N= 957;
Highly exposed N= 1,960, not highly exposed N= 2,114

**Table S2 Participants with "highly exposed" occupations across** Burden of Obstructive Lung Disease (BOLD) follow-up survey **sites.**

| **BOLD site** | **Benin (Sèmè-Kpodji)** | **Estonia (Tartu)** | **Iceland (Reykjavik)** | **India (Mysore)** | **India (Pune)** | **India (Kashmir)** | **Jamaica** | **Kyrgyzstan (Chui)** | **Kyrgyzstan (Naryn)** |
|---|---|---|---|---|---|---|---|---|---|
| Total participants, N | 117 | 405 | 378 | 557 | 751 | 104 | 125 | 549 | 629 |
| Responders to the occupational questionnaire (%) | 115 (98.2) | 391 (96.5) | 376 (99.4) | 246 (44.2) | 684 (91.1) | 52 (50.0) | 83 (65.3) | 345 (62.8) | 339 (53.9) |
| Participants with "not-highly exposed" occupations | 100 (87.0) | 265 (67.8) | 184 (48.9) | 184 (74.8) | 64 (9.3) | 9 (7.4) | 11 (14.3) | 225 (44.6) | 5 (1.5) |
| Participants with "highly exposed" occupations | 14 (13.0) | 126 (32.2) | 192 (51.1) | 62 (25.2) | 620 (90.7) | 43 (82.6) | 72 (86.7) | 120 (55.4) | 334 (98.5) |
| **Occupational exposure to inorganic dusts (%)** | **5 (35.7)** | **48 (38.1)** | **155 (80.7)** | **33 (53.2)** | **63 (10.2)** | **0 (0.0)** | **17 (23.6)** | **27 (14.1)** | **16 (4.8)** |
| Hard rock mining | 0 | 0 | 6 | 0 | 11 | 0 | 0 | 0 | 0 |
| Coal mining | 1 | 4 | 0 | 0 | 0 | 0 | 0 | 4 | 0 |
| Sandblasting | 3 | 0 | 34 | 0 | 30 | 0 | 0 | 0 | 3 |
| Working with asbestos | 0 | 5 | 11 | 0 | 1 | 0 | 0 | 0 | 0 |
| Construction | 0 | 37 | 102 | 33 | 17 | 0 | 17 | 21 | 13 |
| Cement manufacturing | 1 | 2 | 2 | 0 | 4 | 0 | 0 | 2 | 0 |
| Charcoal mining (site-specific) | 0 | 0 | 0 | 0 | 0 | 0 | 0 | 0 | 0 |
| **Occupational exposures to organic dusts (%)** | **7 (50.0)** | **39 (31.0)** | **100 (52.1)** | **23 (37.1)** | **592 (95.5)** | **43 (91.4)** | **25 (34.7)** | **84 (44.0)** | **69 (20.7)** |
| Farming | 2 | 24 | 83 | 23 | 580 | 43 | 19 | 74 | 64 |
| Flour, feed, grain milling or baking | 5 | 13 | 16 | 0 | 9 | 0 | 5 | 9 | 5 |
| Cotton or jute processing | 0 | 2 | 1 | 0 | 3 | 0 | 1 | 1 | 0 |
| **Occupational exposure to fumes (%)** | **2 (13.3)** | **47 (37.3)** | **69 (35.9)** | **0 (0.0)** | **32 (5.2)** | **0 (0.0)** | **5 (6.9)** | **4 (2.1)** | **8 (2.4)** |
| Foundry or steel milling | 0 | 15 | 16 | 0 | 17 | 0 | 0 | 0 | 0 |
| Welding | 2 | 9 | 31 | 0 | 6 | 0 | 4 | 1 | 4 |
| Waste recycling | 0 | 1 | 3 | 0 | 0 | 0 | 0 | 0 | 0 |
| Firefighting | 0 | 5 | 2 | 0 | 0 | 0 | 1 | 0 | 2 |
| Chemical or plastic manufacturing | 0 | 17 | 17 | 0 | 9 | 0 | 0 | 3 | 2 |
| **Occupational exposure to cleaning products (%)** | **0 (0.0)** | **24 (19.0)** | **65 (33.8)** | **4 (6.5)** | **6 (18.8)** | **0 (0.0)** | **21 (29.2)** | **10 (5.2)** | **22 (6.6)** |

| BOLD site | Benin (Sèmè-Kpodji) | Estonia (Tartu) | Iceland (Reykjavik) | India (Mysore) | India (Pune) | India (Kashmir) | Jamaica | Kyrgyzstan (Chui) | Kyrgyzstan (Naryn) |
|---|---|---|---|---|---|---|---|---|---|
| Cleaning | 0 | 24 | 65 | 4 | 6 | 0 | 21 | 10 | 22 |

**Table S2. Participants with "highly exposed" occupations across** Burden of Obstructive Lung Disease (BOLD) survey **sites (continued)**

| BOLD site | Malawi (Blantyre) | Morocco (Fes) | Nigeria (Ile-Ife) | Pakistan (Karachi) | Philippines (Nampicuan-Talugtug) | Sudan (Khartoum) | Sweden (Uppsala) | Tunisia (Sousse) |
|---|---|---|---|---|---|---|---|---|
| Total participants, N | 379 | 78 | 556 | 259 | 482 | 57 | 431 | 279 |
| Responders to the occupational questionnaire (%) | 181 (47.8) | 40 (51.3) | 455 (81.8) | 115 (44.4) | 313 (64.9) | 44 (77.2) | 233 (54.1) | 225 (80.1) |
| Participants with "not-highly exposed" occupations | 45 (24.9) | 27 (67.5) | 351 (77.1) | 93 (80.9) | 96 (30.7) | 22 (50.0) | 148 (63.5) | 145 (64.4) |
| Participants with "highly exposed" occupations | 136 (75.1) | 13 (32.5) | 104 (22.9) | 22 (19.1) | 217 (69.3) | 22 (50.0) | 85 (36.6) | 80 (35.6) |
| **Occupational exposure to inorganic dusts (%)** | **76 (55.9)** | **11 (84.6)** | **21 (20.2)** | **7 (31.8)** | **39 (18.0)** | **6 (27.3)** | **36 (42.3)** | **11 (13.7)** |
| Hard rock mining | 2 | 0 | 4 | 0 | 0 | 2 | 2 | 1 |
| Coal mining | 1 | 0 | 0 | 0 | 1 | 0 | 0 | 0 |
| Sandblasting | 0 | 0 | 3 | 0 | 0 | 1 | 1 | 0 |
| Working with asbestos | 0 | 0 | 0 | 0 | 0 | 1 | 8 | 0 |
| Construction | 15 | 10 | 13 | 7 | 38 | 2 | 23 | 10 |
| Cement manufacturing | 0 | 1 | 1 | 0 | 0 | 0 | 2 | 0 |
| Charcoal mining (site-specific) | 58 | 0 | 0 | 0 | 0 | 0 | 0 | 0 |
| **Occupational exposures to organic dusts (%)** | **72 (39.8)** | **5 (38.5)** | **73 (70.2)** | **5 (22.7)** | **193 (88.9)** | **17 (77.3)** | **31 (36.5)** | **29 (36.3)** |
| Farming | 71 | 4 | 64 | 3 | 186 | 12 | 27 | 1 |
| Flour, feed, grain milling or baking | 1 | 1 | 7 | 0 | 7 | 4 | 4 | 1 |
| Cotton or jute processing | 0 | 0 | 2 | 2 | 0 | 1 | 0 | 27 |

| BOLD site | Malawi (Blantyre) | Morocco (Fes) | Nigeria (Ile-Ife) | Pakistan (Karachi) | Philippines (Nampicuan-Talugtug) | Sudan (Khartoum) | Sweden (Uppsala) | Tunisia (Sousse) |
|---|---|---|---|---|---|---|---|---|
| **Occupational exposure to fumes (%)** | **2 (1.1)** | **1 (7.7)** | **4 (3.8)** | **5 (22.7)** | **6 (2.8)** | **1 (4.5)** | **43 (50.6)** | **9 (11.3)** |
| Foundry or steel milling | 1 | 0 | 2 | 1 | 1 | 0 | 5 | 0 |
| Welding | 1 | 0 | 1 | 2 | 2 | 0 | 15 | 4 |
| Waste recycling | 0 | 0 | 1 | 0 | 1 | 0 | 2 | 0 |
| Firefighting | 0 | 0 | 0 | 0 | 0 | 0 | 7 | 0 |
| Chemical or plastic manufacturing | 0 | 1 | 0 | 2 | 2 | 1 | 14 | 5 |
| **Occupational exposure to cleaning products (%)** | **2 (1.1)** | **0 (0.0)** | **8 (7.7)** | **7 (31.8)** | **8 (3.7)** | **1 (4.5)** | **18 (21.2)** | **0 (0.0)** |
| Cleaning | 2 | 0 |  | 7 | 8 | 1 | 18 | 0 |

**Table S3. Associations between "highly exposed" occupations and respiratory outcomes over time across LMIC sites only**

| | Lung function outcomes | | Respiratory symptoms outcomes | | |
|---|---|---|---|---|---|
| | **N exposed** | **β Coefficient *(95% CI)**** | | **N Exposed** | **RR (95%CI)** |
| **$FEV_1$/FVC** | | | **Dyspnoea** | | |
| Overall | 924 | -0.68 (-1.33, -0.03) | Overall | 183 | 1.05 (0.65, 1.68) |
| Males | 525 | -0.87(-1.81, 0.06) | Males | 74 | 0.89 (0.28, 1.58) |
| Females | 399 | -0.43 (-1.33, 0.47) | Females | 109 | 1.34 (0.78, 2.30) |
| Never smokers | 665 | -0.43 (-1.22, 0.34) | Never smokers | 145 | 1.42 (0.83, 2.41) |
| Ever smokers | 250 | -1.30 (-2.55, -0.02) | Ever smokers | 38 | - |
| **$FEV_1$** | | | **Wheeze** | | |
| Overall | 924 | -0.01 (-0.06, 0.04) | Overall | 176 | 1.44 (1.09, 1.92) |
| Males | 525 | 0.02 (-0.05, 0.09) | Males | 91 | 1.28 (0.90, 1.82) |
| Females | 399 | -0.06(-0.12, 0.01) | Females | 85 | 1.85 (1.27, 2.69) |
| Never smokers | 665 | -0.035(-0.10, 0.01) | Never smokers | 105 | 1.52 (1.02, 2.29) |
| Ever smokers | 250 | 0.06 (-0.03, 0.15) | Ever smokers | 71 | 1.45 (0.97, 2.19) |
| **FVC** | | | **Chronic cough** | | |
| Overall | 924 | 0.02 (-0.04, 0.07) | Overall | 51 | 1.01 (0.76, 1,56) |
| Males | 525 | 0.06 ( -0.02, 0.15) | Males | 32 | 1.42 (0.69, 2.92) |
| Females | 399 | -0.06 (-0.14, 0.02) | Females | 19 | - |
| Never smokers | 665 | -0.04 (-0.11, 0.02) | Never smokers | 27 | - |
| Ever smokers | 250 | 0.13 (-0.02, 0.24) | Ever smokers | 24 | - |
| **CAO** | | | **Chronic phlegm** | | |
| Overall | 49 | - | Overall | 48 | 0.90 (0.56, 1.46) |
| Males | 33 | - | Males | 28 | 0.96 (0.49, 1.89) |
| Females | 16 | - | Females | 20 | - |
| Never smokers | 21 | - | Never smokers | 26 | - |
| Ever smokers | 28 | - | Ever smokers | 22 | - |

**Table S4. Associations between occupational exposures (ALOHA+ JEM) and respiratory outcomes over time across LMIC sites only**

| | $FEV_1$/FVC | | $FEV_1$ | | FVC | | $FEV_1$/FVC <LLN | |
|---|---|---|---|---|---|---|---|---|
| ***ALOHA+ JEM agent*** | ***N*** | ***β* (95% CI)** | ***N*** | ***β* (95% CI)** | ***N*** | ***β* (95% CI)** | **N** | **RR (95% CI)** |
| **VGDF** | | | | | | | | |
| Low (<68 EU-years) | 295 | -0.74 (-1.82, 0.34) | 295 | -0.02 (-0.09, 0.05) | 295 | 0.01 (-0.09, 0.08) | 5 | 0.98 -(0.51, 1.86) |
| Moderate (68-155 EU-years) | 364 | -1.98 (-3.20, -0.76) | 364 | -0.05 (-0.13, 0.04) | 364 | 0.01 (-0.10, 0.10) | 3 | 1.44 -(0.79, 2.60) |
| High (≥156 EU years) | 78 | -2.91 (-4.79, -1.04) | 78 | -0.04 (-0.17, 0.08) | 78 | -0.02 (-0.14, 0.17) | 0 | 1.68 -(0.77, 3.66) |
| **All pesticides** | | | | | | | | |
| Low (≤147 EU-years) | 202 | -1.44 (-2.69, -0.19) | 202 | -0.03 (-0.11, 0.06) | 202 | 0.01 (-0.11, 0.10) | 17 | 1.36 (0.78, 2.36) |
| Moderate (148-179 EU-years) | 176 | -1.48 (-2.95, -0.02) | 176 | -0.01 (-0.11, 0.09) | 176 | 0.02 (-0.10, 0.15) | 15 | 1.18 (0.62, 2.22) |
| High (≥180 EU-years) | 51 | -2.99 (-5.12, -0.86) | 51 | 0.01 (-0.13, 0.15) | 51 | 0.10 (-0.07, 0.27) | 7 | 1.46 (0.63, 3.40) |

| | | | | | | | | |
|---|---|---|---|---|---|---|---|---|
| **All solvents** | | | | - | | | | |
| Low (<38 EU-years) | 101 | -1.07 (-2.56, 0.42) | 101 | -0.03 (-0.12, 0.07) | 101 | -0.01 (-0.12, 0.12) | 9 | 1.19 (0.61, 2.37) |
| Moderate (38-45 EU-years) | 74 | -1.14 (-2.91, 0.64) | 74 | -0.05 (-0.17, 0.07) | 74 | -0.02 (-0.17, 0.12) | 6 | 0.78 (0.34, 1.79) |
| High (≥46 EU-years) | 25 | -0.94 (-3.75, 1.86) | 25 | -0.10 (-0.29, 0.08) | 25 | -0.09 (-0.32, 0.13) | 5 | - |
| **Metals** | | | | | | | | |
| Low (<31 EU-years) | 40 | -1.09 (-3.30, 1.15) | 40 | 0.08 (-0.06, 0.22) | 40 | 0.16 (-0.02, 0.33) | 5 | - |
| Moderate (31 - 44 EU-years) | 19 | -0.78 (-3.95, 0.31) | 19 | -0.05 (-0.25, 0.16) | 19 | -0.03 (-0.28, 0.22) | 3 | - |
| High (≥45 EU-years) | 6 | - | - | | 6 | | 0 | - |
| **Respiratory symptoms** | | | | | | | | |
| | **Wheeze** | | **Chronic cough** | | **Chronic phlegm** | | **Dyspnoea** | |
| | **N** | **RR (95% CI)** | **N** | **RR (95% CI)** | **N** | **RR (95% CI)** | **N** | **RR (95% CI)** |
| **VDGF** | | | | | | | | |

| | | | | | | | | |
|---|---|---|---|---|---|---|---|---|
| Low<br>(<68 EU-years) | 43 | 1.17<br>(0.75, 1.84) | 15 | 1.09<br>(0.53, 1.66) | 12 | 0.81<br>(0.40, 1.72) | 29 | 0.90<br>(0.47, 1.71) |
| Moderate<br>(68-155 EU-years) | 52 | 1.35<br>(0.87, 2.10) | 10 | 0.57<br>(0.20, 1.29) | 9 | 0.34<br>(0.12, 0.96) | 51 | 0.92<br>(0.37, 2.37) |
| High<br>(≥156 EU years) | 17 | 1.65<br>(0.89, 3.06) | 5 | - | 4 | - | 26 | 0.99<br>(0.22, 4.41) |
| **All pesticides** | | | | | | | | |
| Low<br>(≤147 EU-years) | 33 | 1.31<br>(0.85, 2.20) | 4 | - | 6 | - | 24 | 1.76<br>(0.76, 4.05) |
| Moderate<br>(148-179 EU-years) | 27 | 1.54<br>(1.05, 2.15) | 3 | - | 5 | - | 33 | 2.38<br>(0.90. 6.34) |
| High<br>(≥180 EU-years) | 15 | 2.20<br>(1.17, 4.14) | 5 | - | 3 | - | 23 | 1.90<br>(0.52, 6.90) |
| **All solvents** | | | | | | | | |
| Low<br>(<38 EU-years) | 14 | 1.13<br>(0.59, 2.16) | 2 | - | 2 | - | 6 | - |
| Moderate<br>(38-45 EU-years) | 17 | 1.42<br>(0.78, 2.61) | 1 | - | 4 | - | 2 | - |
| High<br>(≥46 EU-years) | 6 | - | 0 | - | 0 | - | 3 | - |

| | | | | | | | | |
|---|---|---|---|---|---|---|---|---|
| **Metals** | | | | | | | | |
| Low (<31 EU-years) | 5 | - | 4 | - | 3 | - | 2 | - |
| Moderate (31 - 44 EU-years) | 6 | - | 1 | - | 1 | - | 1 | - |
| High (≥45 EU-years) | 1 | - | 0 | - | 0 | - | 1 | - |

**Table S5. Associations between occupational exposures (ALOHA+ JEM) and respiratory outcomes over time in sites with a response rate >50% in the OSCAR questionnaire.**

| | **$FEV_1$/FVC** | | **$FEV_1$** | | **FVC** | | **$FEV_1$/FVC <LLN** | |
|---|---|---|---|---|---|---|---|---|
| ***ALOHA+ JEM agent*** | ***N*** | ***β* (95% CI)** | ***N*** | ***β* (95% CI)** | ***N*** | ***β* (95% CI)** | **N** | **RR (95% CI)** |
| **VGDF** | | | | | | | | |
| Low (<68 EU-years) | 284 | 0.21 (-0.79, 1.22) | 284 | 0.03 (-0.03, 0.10) | 284 | 0.01 (-0.07, 0.09) | 20 | 1.20 (0.69, 2.41) |
| Moderate (68-155 EU-years) | 275 | -1.06 (-2.38, 0.25) | 275 | -0.01 (-0.09, 0.08) | 275 | 0.01 (-0.09, 0.12) | 23 | 2.00 (1.07, 3.74) |
| High (≥156 EU years) | 73 | -1.14 (-3.02, 0.75) | 73 | -0.01 (-0.13, 0.11) | 73 | -0.01 (-0.16, 0.14) | 7 | - |

| | | | | | | | | |
|---|---|---|---|---|---|---|---|---|
| **All pesticides** | | | | | | | | |
| Low (≤147 EU-years) | 122 | -1.44 (-3.10, 0.22) | 122 | -0.01 (-0.12, 0.10) | 122 | 0.03 (-0.11, 0.16) | 6 | - |
| Moderate (148-179 EU-years) | 149 | -1.01 (-2.75, 0.73) | 149 | -0.02 (-0.13, 0.10) | 149 | 0.01 (-0.14, 0.15) | 11 | - |
| High (≥180 EU-years) | 44 | -1.91 (-4.21, 0.40) | 44 | 0.03 (-0.12, 0.17) | 44 | 0.08 (-0.11, 0.27) | 4 | - |
| **All solvents** | | | | | | | | |
| Low (<38 EU-years) | 61 | -0.19 (-1.88, 1.49) | 61 | 0.03 (-0.07, 0.14) | 61 | 0.03 (-0.10, 0.17) | 4 | - |
| Moderate (38-45 EU-years) | 59 | -0.34 (-2.14, 1.47) | 59 | -0.05 (-0.16, 0.07) | 59 | -0.06 (-0.21, 0.08) | 2 | - |
| High (≥46 EU-years) | 30 | 0.50 (-1.94, 2.94) | 30 | 0.03 (-0.10, 0.20) | 30 | 0.04 (-0.15, 0.23) | 3 | - |
| **Metals** | | | | | | | | |
| Low (<31 EU-years) | 21 | -0.02 (-2.87, 2.83) | 21 | 0.04 (-0.14, 0.22) | 21 | 0.05 (-0.17, 0.27) | 0 | - |
| Moderate (31 - 44 EU- | 25 | -2.54 | 25 | -0.01 | 25 | 0.09 | 3 | - |

| | | | | | | | | |
|---|---|---|---|---|---|---|---|---|
| years) | | (-5.26, 0.18) | | (-0.18, 0.16) | | (-0.13, 0.30) | | |
| High (≥45 EU-years) | 7 | - | 7 | - | 7 | | 1 | - |
| **Respiratory symptoms** | | | | | | | | |
| | **Wheeze** | | **Chronic cough** | | **Chronic phlegm** | | **Dyspnoea** | |
| | **N** | **RR (95% CI)** | **N** | **RR (95% CI)** | **N** | **RR (95% CI)** | **N** | **RR (95% CI)** |
| **VDGF** | | | | | | | | |
| Low (<68 EU-years) | 73 | 0.98 (0.68, 1.43) | 37 | 1.06 (0.63, 1.78) | 20 | 0.49 (0.425, 0.97) | 25 | 1.19 (0.50, 2.84) |
| Moderate (68-155 EU-years) | 52 | 1.21 (0.79, 1.83) | 13 | 0.69 (0.31, 1.51) | 10 | 0.54 (0.23, 1.28) | 49 | 1.69 (0.44, 6.51) |
| High (≥156 EU years) | 17 | 1.55 (0.82, 2.94) | 5 | - | 5 | - | 26 | 4.15 (0.49, 9.25) |
| **All pesticides** | | | | | | | | |
| Low (≤147 EU-years) | 20 | 1.12 (0.62, 2.02) | 3 | - | 4 | - | 20 | 1.81 (0.93, 4.05) |
| Moderate (148-179 EU-years) | 23 | 1.70 (1.01, 2.86) | 1 | - | 2 | - | 32 | 2.22 (1.53. 6.34) |

| | | | | | | | | |
|---|---|---|---|---|---|---|---|---|
| High (≥180 EU-years) | 13 | 2.16 (1.11, 4.22) | 4 | - | 3 | - | 21 | 3.37 (0.52, 9.90) |
| **All solvents** | | | | | | | | |
| Low (<38 EU-years) | 22 | 1.12 (0.62, 2.02) | 9 | 0.63 (0.23, 1.73) | 10 | 1.19 (0.50, 2.86) | 8 | 1.07 (0.43, 2.34) |
| Moderate (38-45 EU-years) | 25 | 1.70 (1.01, 2.86) | 12 | 1.39 (0.67, 2.92) | 7 | 0.90 (0.34, 2.39) | 7 | 0.69 (0.21, 1.90) |
| High (≥46 EU-years) | 4 | - | 4 | - | 1 | - | 1 | - |
| **Metals** | | | | | | | | |
| ow (<31 EU-years) | 7 | - | 4 | - | 3 | - | 1 | - |
| Moderate (31 - 44 EU-years) | 8 | - | 4 | - | 3 | - | 2 | - |
| High (≥45 EU-years) | 3 | - | 2 | - | 0 | - | 0 | - |

**Fig S1. Participants who responded to the occupational questionnaire in BOLD 2 by site**

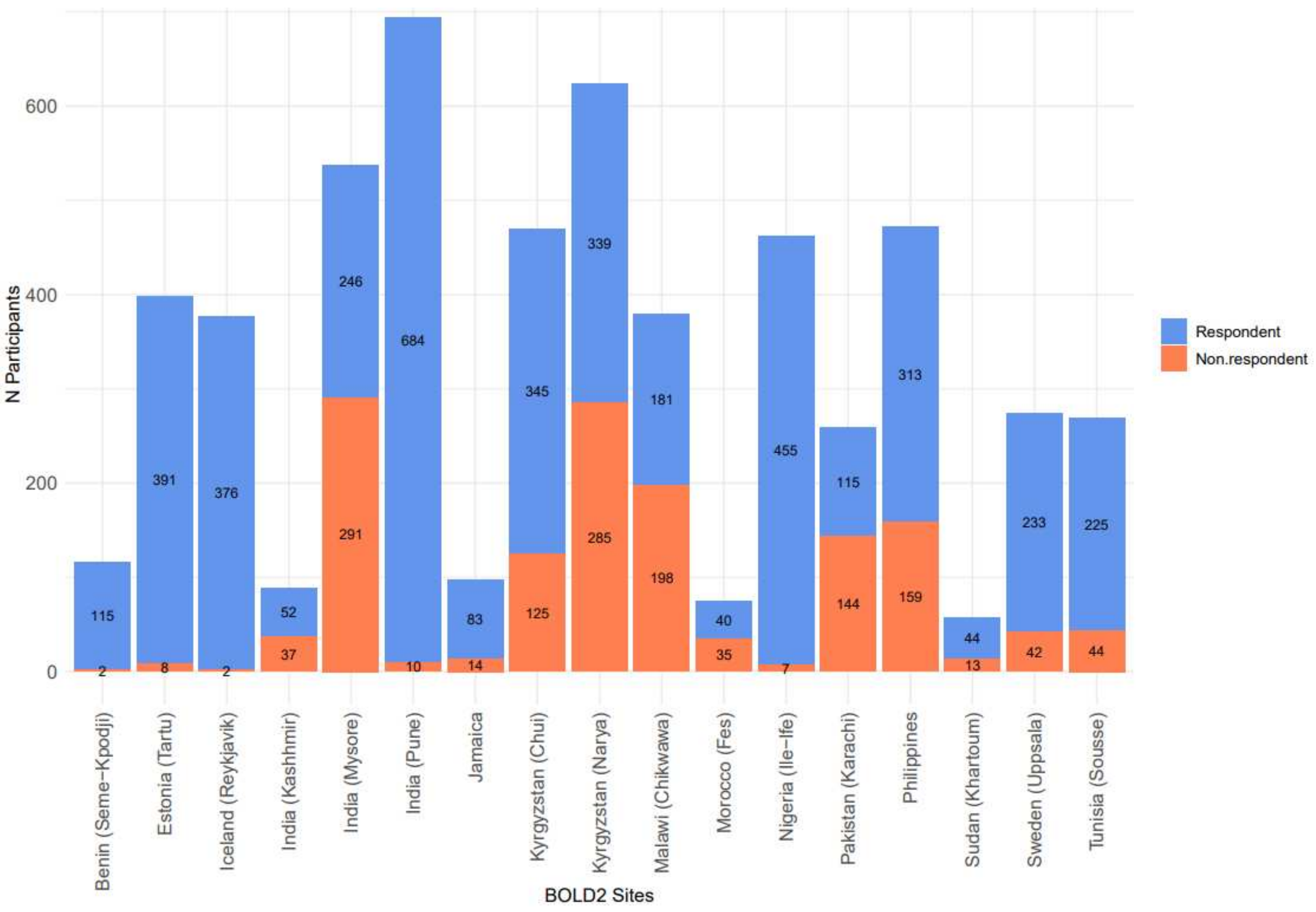

**Figure S2. Lung function changes in participants with occupational exposures over time in the BOLD study**

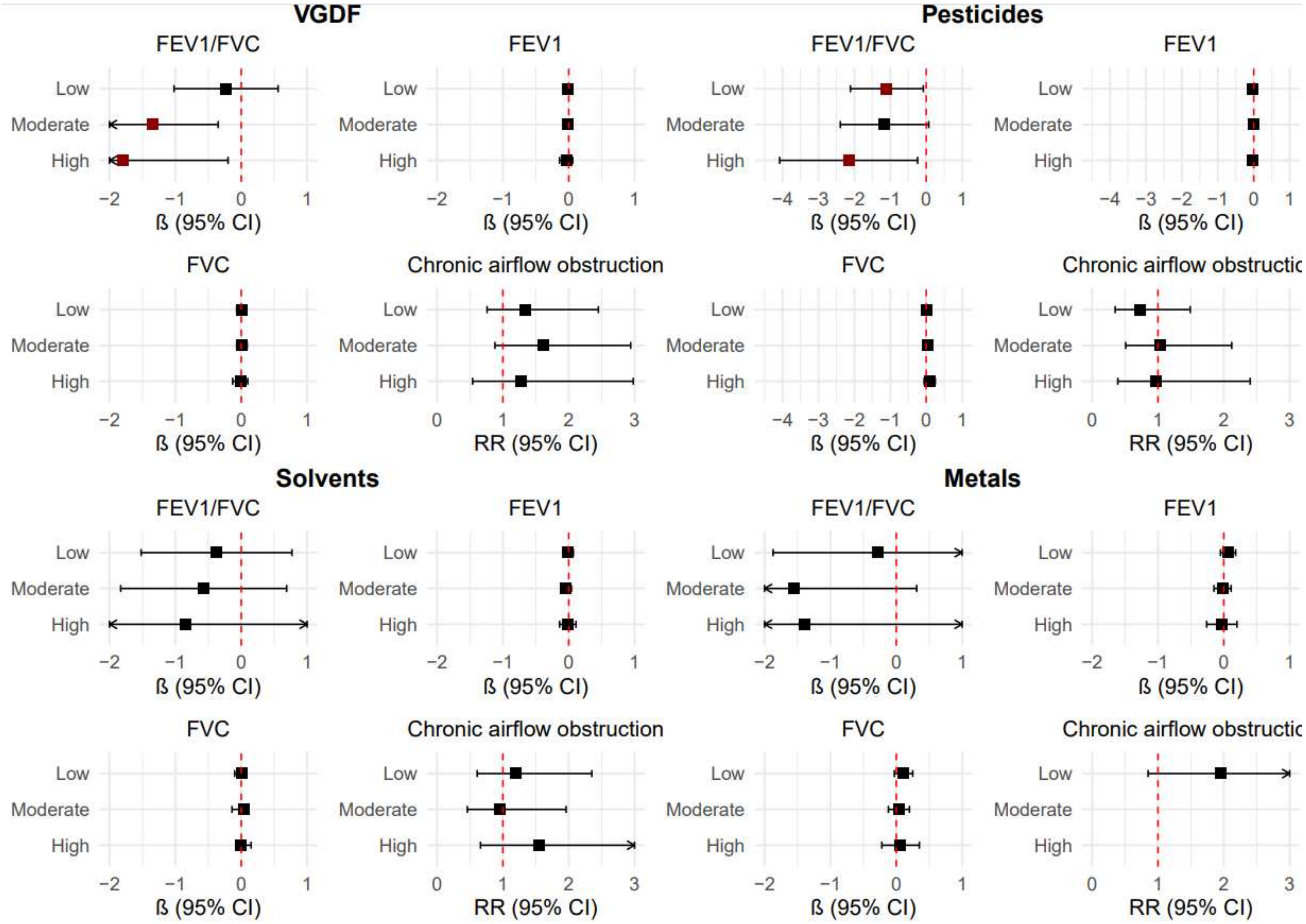

**Fig S3. Respiratory symptom risk in participants with occupational exposures over time in the BOLD study**

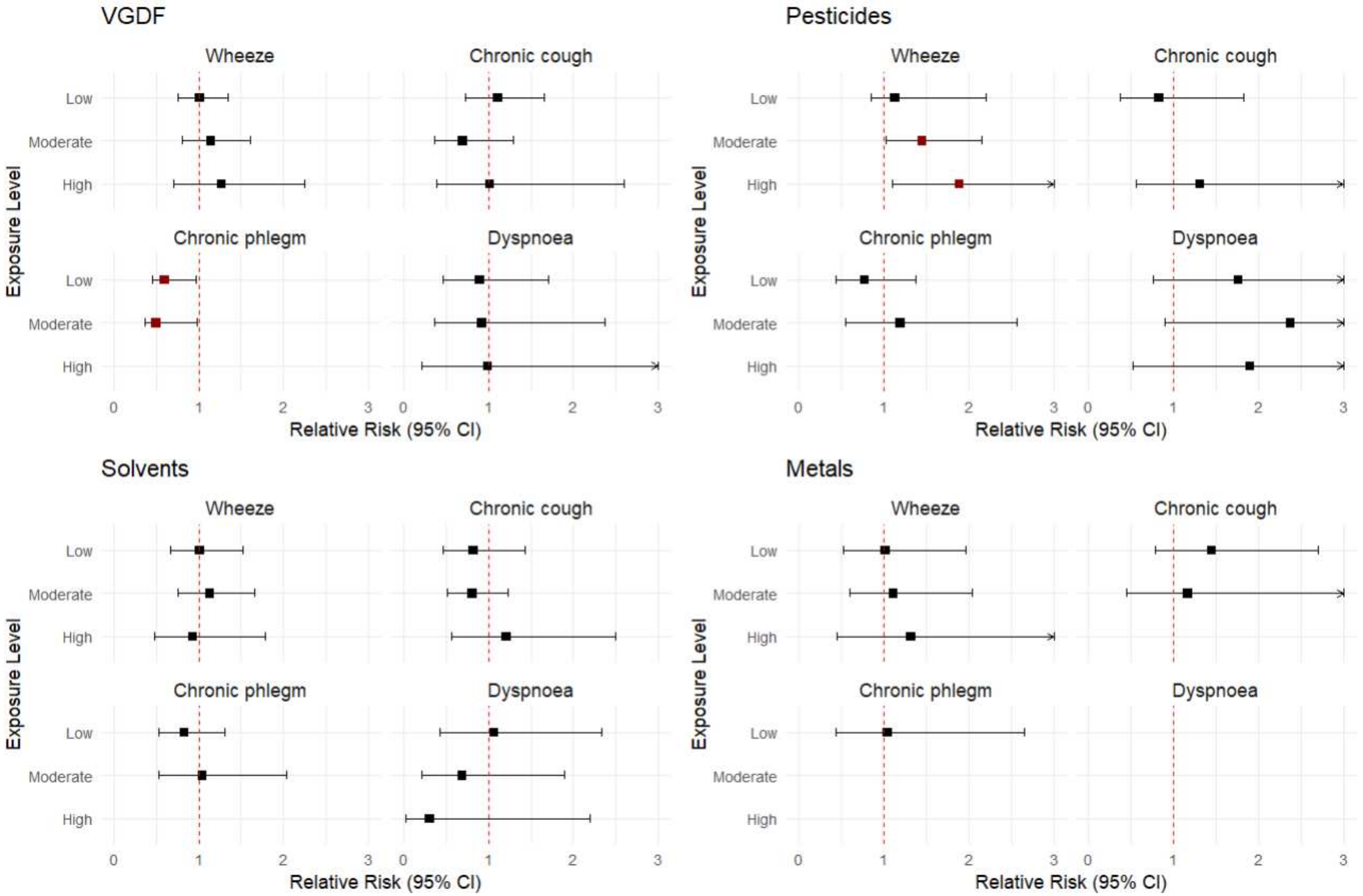

**Fig S4. Dose-response association between VGDF exposure and respiratory symptoms**

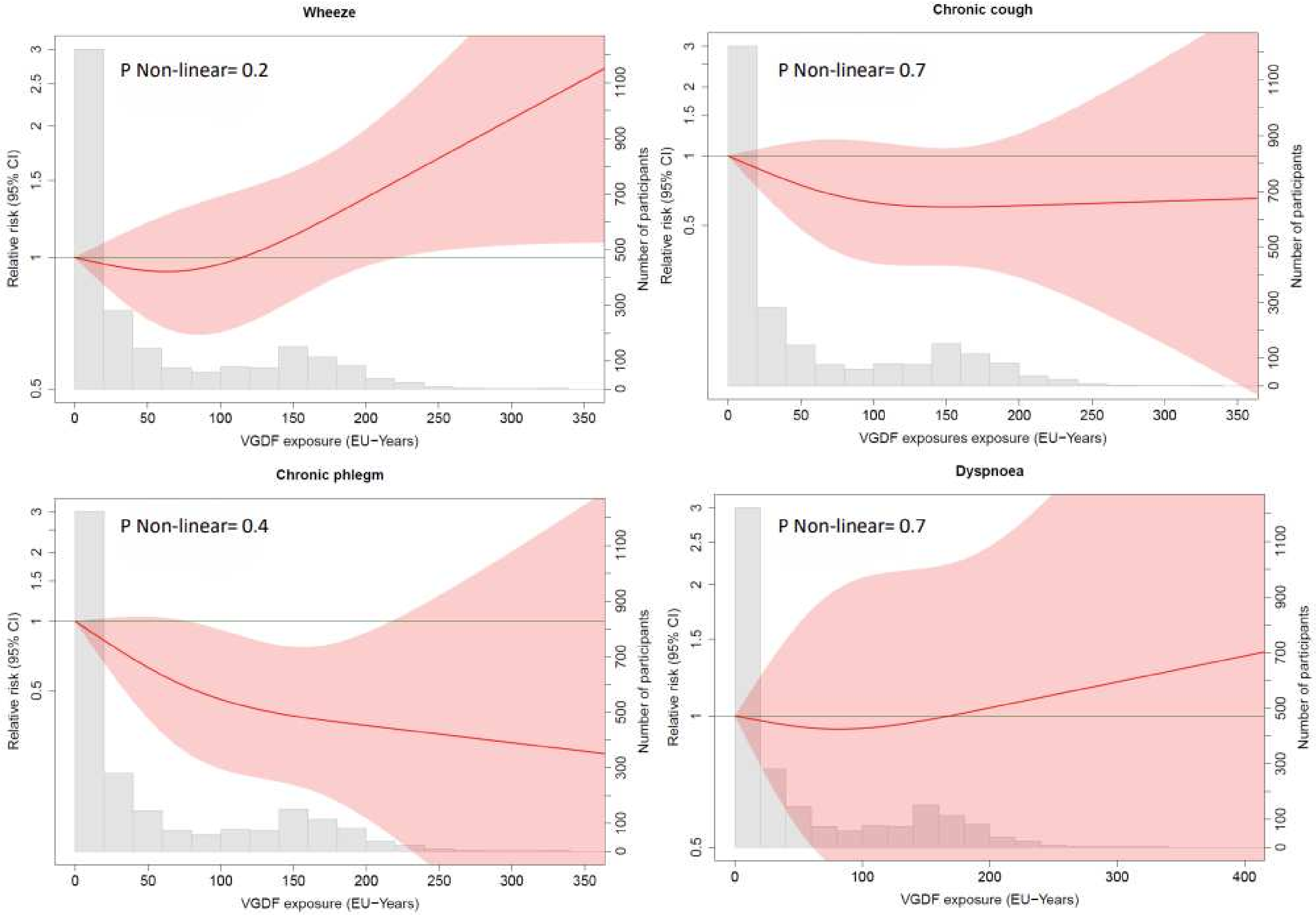

**Fig S5. Dose-response association between pesticides exposure and respiratory symptoms.**

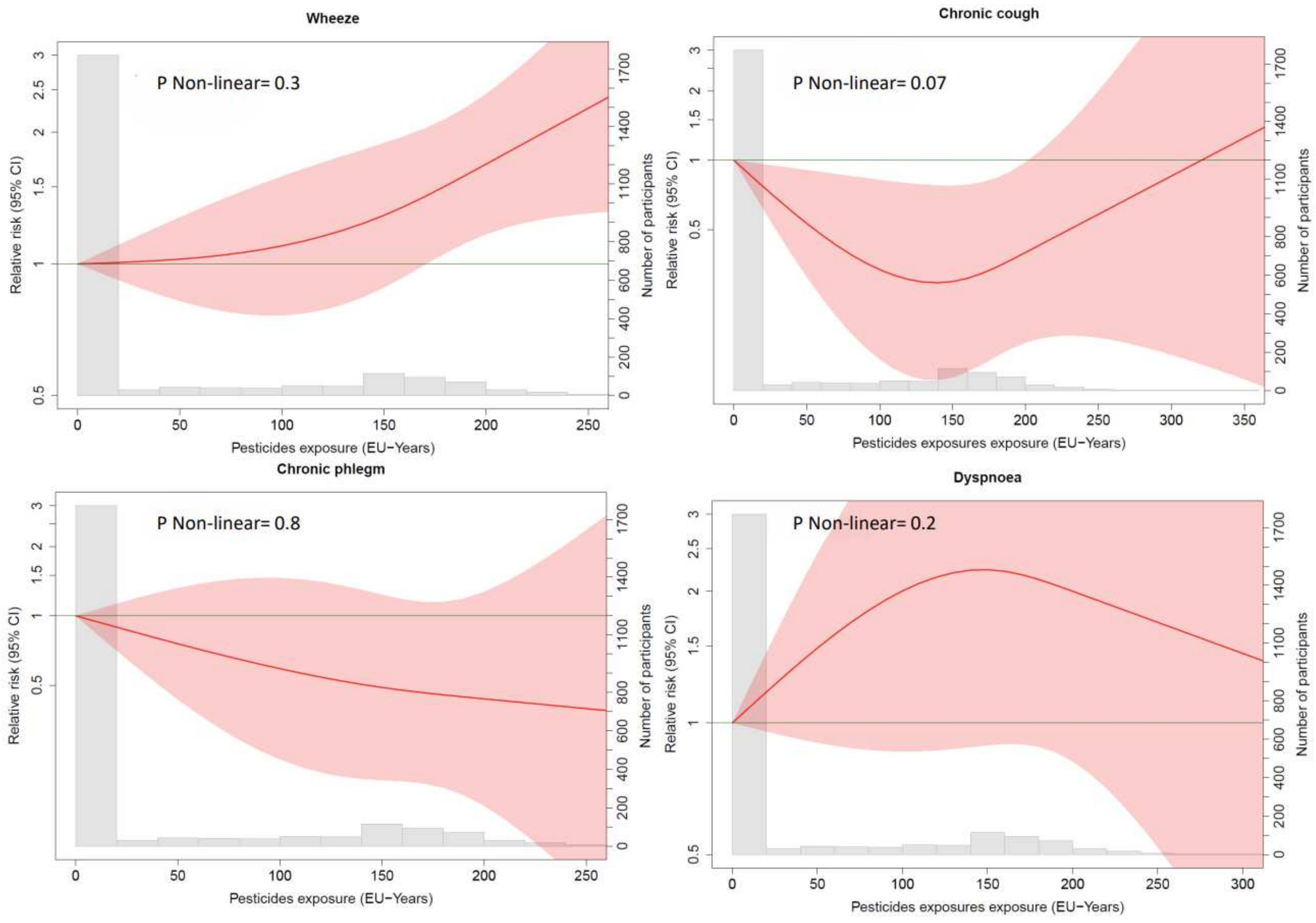

**Fig S6. Dose-response association between solvents exposure and respiratory symptoms.**

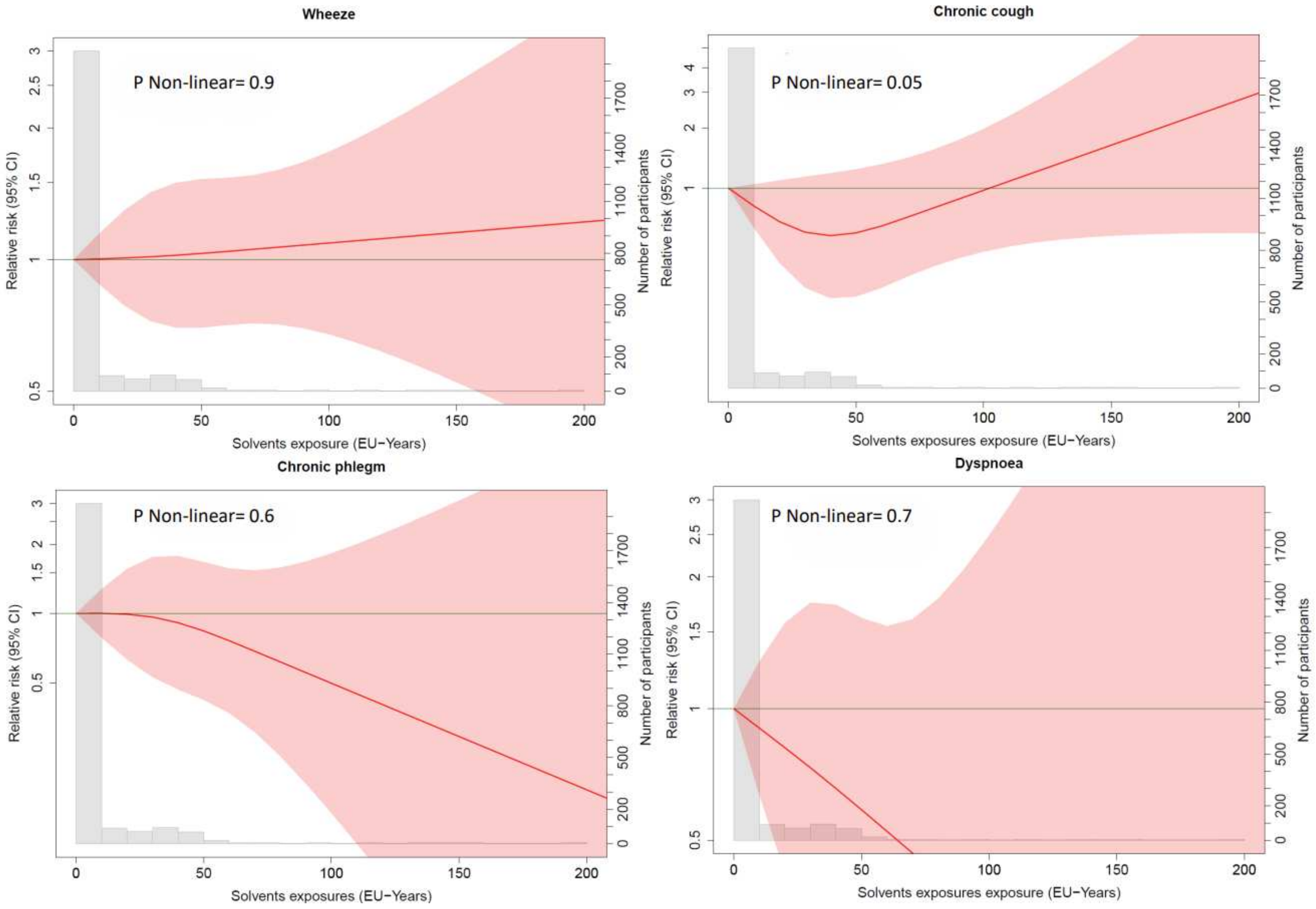

**Fig S7. Dose-response association between metal exposure and respiratory symptoms.**

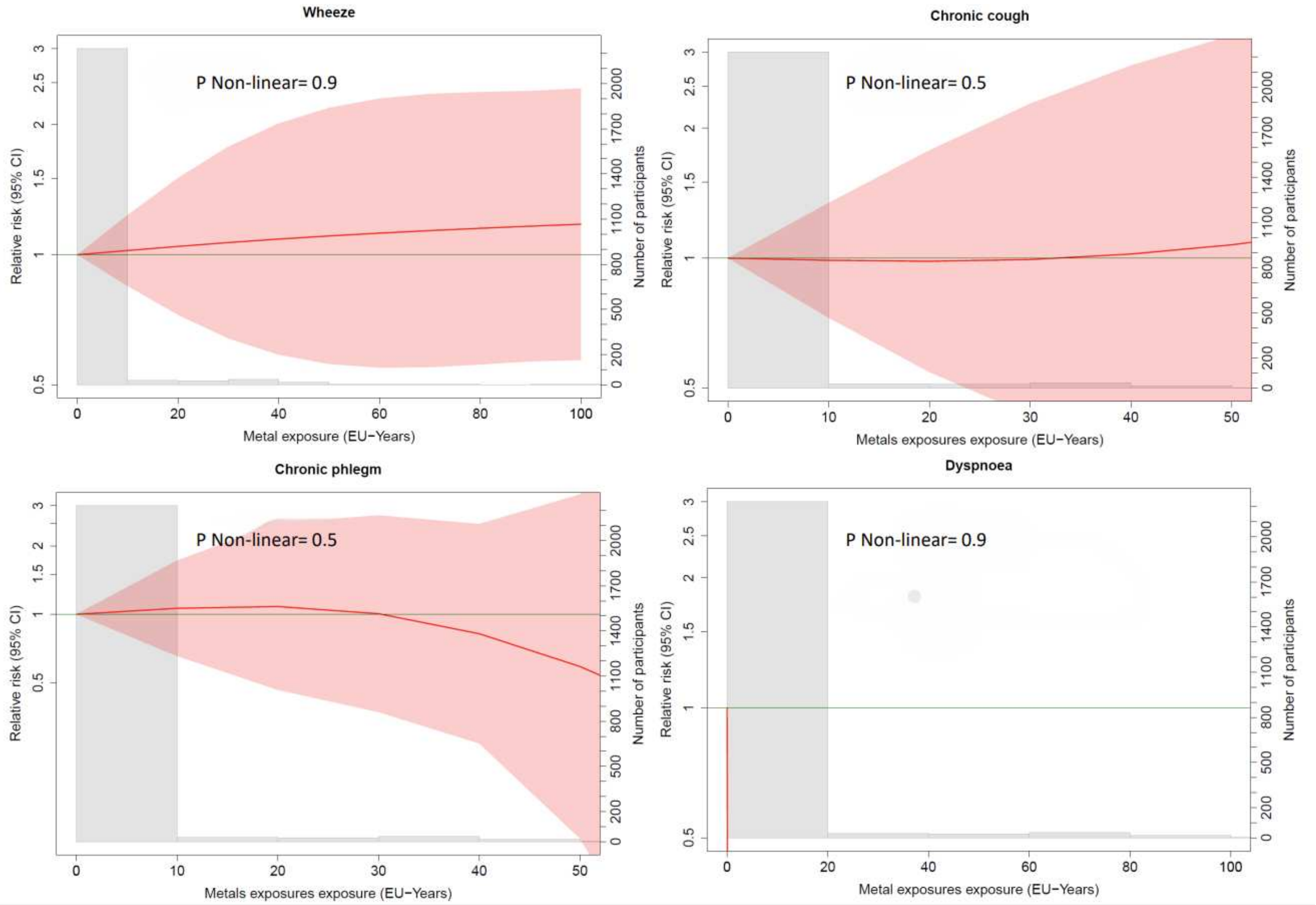

Supplement: Supplementary data [file bmjresp-13-1-s001.pdf]
